# The Transformation Experiment of Frederick Griffith II: Inclusion of Cellular Heredity for the Creation of Novel Microorganisms

**DOI:** 10.3390/bioengineering12050532

**Published:** 2025-05-15

**Authors:** Günter A. Müller

**Affiliations:** 1 Biology and Technology Studies Institute Munich (BITSIM), 80939 Munich, Germany; guenter.al.mueller@t-online.de; Tel.: +49-151-25216987; 2 Institute of Media Sociology, Department of Cultural Sciences, University of Paderborn, 33104 Paderborn, Germany

**Keywords:** bacterial cytoskeleton, bacterial lipoproteins, cyborg bacteria, horizontal or lateral gene transfer, science and technology studies, topological and cellular heredity, transformation specificity

## Abstract

So far, synthetic biology approaches for the construction of artificial microorganisms have fostered the transformation of acceptor cells with genomes from donor cells. However, this strategy seems to be limited to closely related bacterial species only, due to the need for a “fit” between donor and acceptor proteomes and structures. “Fitting” of cellular regulation of metabolite fluxes and turnover between donor and acceptor cells, i.e. cybernetic heredity, may be even more difficult to achieve. The bacterial transformation experiment design 1.0, as introduced by Frederick Griffith almost one century ago, may support integration of DNA, macromolecular, topological, cybernetic and cellular heredity: (i) attenuation of donor *Pneumococci* of (S) serotype fosters release of DNA, and hypothetically of non-DNA structures compatible with subsequent transfer to and transformation of acceptor *Pneumococci* from (R) to (S) serotype; (ii) use of intact donor cells rather than of subcellular or purified fractions may guarantee maximal diversity of the structural and cybernetic matter and information transferred; (iii) “Blending” or mixing and fusion of donor and acceptor *Pneumococci* may occur under accompanying transfer of metabolites and regulatory circuits. A Griffith transformation experiment design 2.0 is suggested, which may enable efficient exchange of DNA as well as non-DNA structural and cybernetic matter and information, leading to unicellular hybrid microorganisms with large morphological/metabolic phenotypic differences and major features compared to predeceding cells. The prerequisites of horizontal gene and somatic cell nuclear transfer, the molecular mechanism of transformation, the machineries for the biogenesis of bacterial cytoskeleton, micelle-like complexes and membrane landscapes are briefly reviewed on the basis of underlying conceptions, ranging from Darwin’s “gemmules” to “stirps”, cytoplasmic and “plasmon” inheritance, “rhizene agency”, “communicology”, “transdisciplinary membranology” to up to Kirschner’s “facilitated variation”.

## 1. Introduction

Ernst-Ludwig Winnacker, the German chemist and pioneer of gene technology, used in 1985 for the preface of his book “Gene und Klone” [1] the following statement of George Bernhard Shaw [2]: “*You see things, and say why? But I dream things that never were, and I say, why not?*”. Apparently, Winnacker shared this dream with Shaw and putatively with other luminaries of early biochemistry, genetics and developmental biology, among them Jacques Loeb [3] (“*Nothing indicates, however, at present that the artificial production of living matter is beyond the possibilities of science…experimental abiogenesis is the goal of biology*”) to up to the very beginnings of genetic engineering, among them Tom Maniatis, Edward F. Fritsch and Joseph Sambrook [4]. According to the latter, the construction de novo of (micro)organisms, which have not existed on the earth so far, in their labs is feasible in principle and only a matter of time, and critically depends on the nucleotide exchange of more or less extended regions of their genomes, exclusively. Their hope and confidence in the potency of recombinant DNA technology subsequently seemed to have been completely fulfilled, with the development of very sophisticated and advanced methods for the partial or total synthesis of complete genomes (for a review, see Refs. [5,6]). However, Winnacker and presumably the majority of biochemists, geneticists, and molecular biologists of that and subsequent periods did not take into consideration the role or agency of the history or origin of a given cell in affecting or determining the fate or future of its successor.

Neverthess, their—admittedly extraordinary—expectations on the creative potential of gene technologists and DNA engineers, sometime called “linear biologists”, for the construction of so far non-existing microorganisms may not go unmet but only if the human as well as non-human (f)actors of macromolecular, topological, cybernetic and cellular, rather than merely of DNA (transformation and) heredity can be delineated and modified according to the technical possibilities as well as the underlying conceptual framework. The latter seems to be achievable—on an unbiased and non-excluding basis—with the aid of Science and Technology Studies (STS) as previously introduced by Donna Harraway [7,8], in general, and the Actor-Network Theory as initially proposed by Bruno Latour [9,10], Michel Callon [11] and John Law [12] as well as the Agential Realism as originally developed by Karen Barad [13,14,15], in particular: “Facilitated variation”, “materialistic organicism”, “rhizene agency”, “communicology” and “transdisciplinary membranology” (see Conclusions) might cause “Cutting Things Together-Apart” for the creation of organisms with major differences and novel building plans compared to their predecessors. The latter, such as genetically modified organisms, in general, and transformed bacteria, in particular, apparently represent only points of attack for biopolitical engagement, which is crucially motivated by their principal predictability, controllability, and manipulability. In a previous report [16], the agencies and (f)actors of bacterial transformation were dealt with for the “natural” creation of prokaryotes. This type of STS study has now been extended to the “artificial” construction of both prokaryotic and unicellular eukaryotic microorganisms.

## 2. The Griffith Transformation Experiment

### 2.1. Previous Designs 1.0 and 1.1

**Figure 1:**
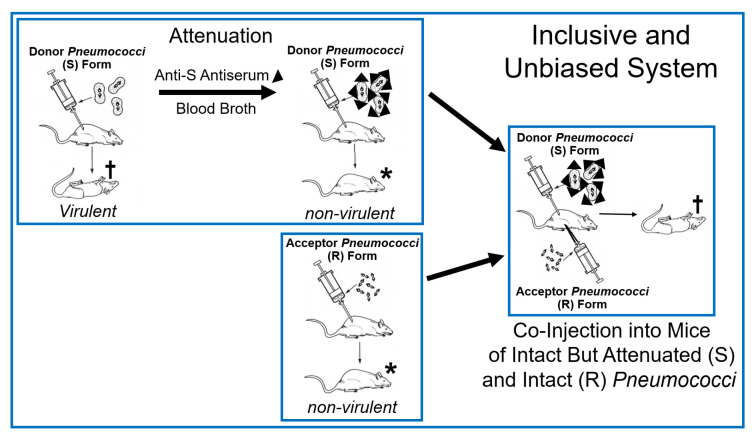
Design of the Griffith transformation experiment 1.0. (S) *Pneumococci* were cultured in the presence of blood/chocolate broth and anti-S antiserum (filled triangles) for their attenuation, which followed two aims: (i) Reduction in the vitality of the (S) donor *Pneumococci* to a level which enabled survival of the mice upon their injection, with accompanying facilitation of detection of virulent *Pneumococci* newly emerging from non-virulent (R) acceptor *Pneumococci* and initially lacking a capsule of the (S) serotype rendering them susceptible to immunological elimination by the host. (ii) Initiation of the release of putative matter of inheritance by the (S) donor *Pneumococci* for subsequent transfer to (R) acceptor *Pneumococci* which led to transformation of *Pneumococci* from the virulent to the non-virulent nature for mice, as indicated by † and *, respectively. These two purposes of the attenuation procedure are presumably linked to one another. Co-injection of the attenuated non-virulent (S) donor *Pneumococci* together with non-virulent (R) acceptor *Pneumococci* caused death of the mice.

The epochal experimental designs, as realized by Frederick Griffith in 1928 [17], are briefly presented to provide a common basis for both their canonical and alternative interpretations. *Pneumococci* (*Diplococcus* or *Streptococcus pneumoniae*) of smooth (S) pathogenic serotypes, which are characterized by the presence of a capsule consisting of variable glycophospholipids, typical O-acetylated lipopolysaccharides (LPS) and lipoproteins (LP) anchored at the peptidoglycan layer. This capsule determines the immunological serotype of *Pneumococci* (for a review, see Refs. [18,19,20]) and mediates eventual evasion from phagocytosis by the immune system of the host (for a review, see Refs. [21,22,23]), leading to the death of normal mice upon injection (virulence). This experimental system turned out to be, per se, inclusive and unbiased (Figure 1).

**Figure 2:**
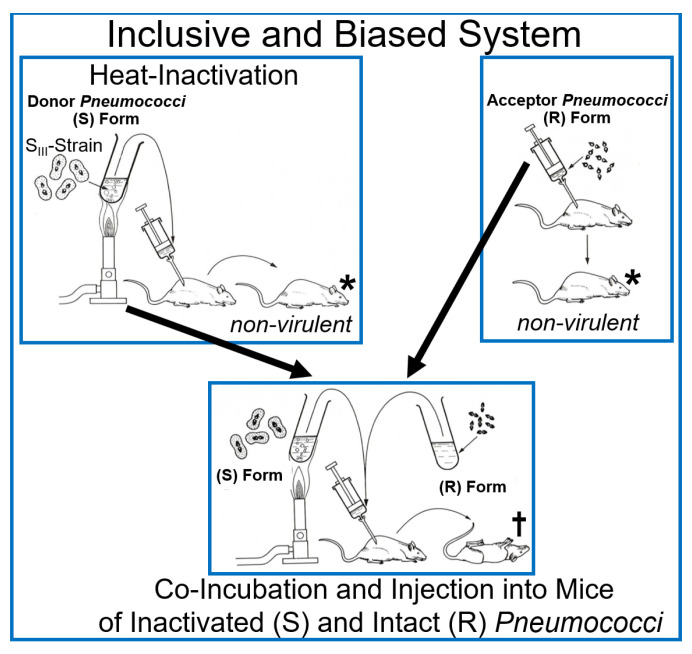
Design of the Griffith transformation experiment 1.1. (S) donor *Pneumococci* were heat-inactivated (80 °C, 2–3 h), leading to their non-virulence upon injection into mice. Their co-injection, together with non-virulent (R) acceptor *Pneumococci*, killed the mice. See Figure 1 for symbols.

At variance, the experimental system subsequently used by Griffith must be interpreted as being (i) biased for the identification of heat-stable matter as the cause for the transfer of the virulence trait and yet; (ii) inclusive with regard to the principal possibility to unravel and discriminate the operation of macromolecular, topological and cellular, in addition of DNA, heredity (Figure 2) (for a review, see Refs. [16,24]).

In the following, this experimental design led to determination of the agency of DNA as the sole hereditary matter by Oswald Avery, Colin MacLeod and Maclyn McCarty with concomitant exclusion of other putative macromolecular, topological, and cybernetic matter and information of cellular heredity, among them proteins and LPS, membranes and organelles, and metabolite fluxes and regulatory circuits, respectively. This canonical view on the Griffith transformation experiment can be found in many outdated [25,26,27], but also current textbooks of genetics and biochemistry.

Certainly, there is no doubt that DNA is required for the switch from the (R) to the (S) phenotype of *Pneumococci*. Moreover, the protease treatment seems to be in conflict with the involvement of proteins, although not immediately of LPS and membranes, as a matter of inheritance, in addition, as first demonstrated by Avery and coworkers [28]. However, from their findings and those of their successors it cannot be excluded that other features of *Pneumococci* (e.g., *Diplococcus* morphology), which have not been used as selection and assay criteria in the course of the transformation experiments following design 1.0 to 1.X, are transferred through non-DNA matter of inheritance, comprising proteins, LPS, and membranes and other macromolecular assemblies, either as separate entities or in concert. Consequently, a modification of the Griffith transformation experiment, termed in the following design 2.0, has recently been proposed to address the question as to whether non-DNA matter may be involved in switching the phenotype of *S. pneumoniae* from avirulent non-pathogenic (R) to the virulent pathogenic (S) [16]. This could be easily assayed under appropriate selection conditions, meaning that there is intercellular transfer of non-DNA matter and/or information between prokaryotes. As a type of “mutagenic” or environmental (f)actor, mechanical stress, such as shearing forces and distortion, could be applied in order to induce the release of putative matter of inheritance from the bacteria, such as plasma membrane (PM) vesicles. The release of vesicles from the PM of Gram-positive bacteria has been amply documented in the past (for a review, see Refs. [29,30,31,32,33]) as well as their involvement in the transfer of surface antigens and receptors from donor to acceptor bacteria (for a review, see Refs. [34,35,36,37]. This apparently provocative possibility has been confirmed in subsequent investigations.

At variance, the possibility of the release of small domains at the PM, constituted by specific subsets of LP, transmembrane proteins, cytoskeletal elements, phospholipids, LPS and other components, which together constitute the so-called “membrane landscapes”, from donor bacteria in response to environmental (f)actors, such as mechanical stress, remains to be demonstrated in the future. The accompanying temporal and spatial reconfiguration, rearrangement, and reorientation of the “membrane landscapes” may result in “re-shaping” or “re-materializing” of a specific phenotype, such as the (R) to the (S) serotype. Following their transfer as so-called micelle-like LP/LPS complexes to and insertion into the PM of acceptor *Pneumococci*, the transferred “membrane landscapes” of the (S) phenotype could trigger the spatial reconfiguration, rearrangement, and reorientation of pre-existing “membrane landscapes”, which so far corresponded to the (R) phenotype of the acceptor bacteria. The molecular mechanism involved seems to resemble the so-called “PM memory” (see below).

Upon further cultivation of the *Pneumococci*, the emergence of colonies displaying the (S) serotype will indicate its stable inheritance. Sequencing of the whole genome from individual colonies of the *Streptococci* will indicate whether some bacteria succeeded in acquiring the (S) serotype in the absence of any mutations in the genome. Thereby, the new (S) serotype would be stably maintained in the next generations of bacteria. A synopsis of the various experimental designs of the Griffith transformation experiment including their interpretation with regard to the entities and principles transferred, the theory and type of inheritance as well as the underlying intention, which had already been carried out (1.0–1.3) and those which remain to be performed in the future (1.4–1.X; for a review, see Ref. [16]) is presented (Table 1).

In conclusion, the experimental system of bacterial transformation introduced by Griffith in 1928 [17] opened the path towards the identification of the underlying transforming principle. A closer look (see Ref. [16]) revealed that his initial design left open the possibility of alternative interpretations: In addition to DNA, LPS and LP arranged in micelle-like complexes or PM vesicles could be regarded as non-DNA matter of inheritance which causes transfer of structural (e.g., membranes) and cybernetic (e.g., regulatory circuits) information from donor to acceptor bacteria. The focus on the inheritance of minor features and small differences may have favored the “DNA-centric”, “hard”, “excluding”, biased vs. an “extended”, “soft”, “inclusive”, unbiased view. Similarly, the experimental designs of phage infection and chromosome transfer, first introduced by Alfred Hershey and Martha Chase [38] and Edward Tatum and Joshua Lederberg [39], respectively, had also been interpreted in a “DNA-centric” fashion [16]. The “extended” view of inheritance led to a shift from transfer of DNA, genes and nucleus matter to macromolecules to structures and topologies to regulatory circuits and metabolism to finally cells as “the units” of heredity and might be helpful for the creation of novel microorganisms which goes beyond “mere” transformation or horizontal gene transfer.

### 2.2. Early Findings on Transformation

#### 2.2.1. Mechanisms of Bacteria

As far as it is known, bacterial transformation does not occur in all, but in a large number of bacterial species, in addition to *Pneumococcus*, also in e.g., *Bacillus subtilis*, *Haemophilus influenzae*, *Neisseria*, *Rhizobium*, and *Acinetobacter* (for a review, see Ref. [40]). In transformation experiments, in addition to serological traits already used by Avery and coworkers [28], mainly auxotrophies have been used. For example, tryptophan-needing cells of *B. subtilis* can be treated with DNA of a tryptophan-independent strain. After spreading on minimal medium, prototrophic transformants were obtained. Resistance to antimetabolites and antibiotics was also a popular feature in many transformation attempts. The conditions, that must be met in order to successfully transfer such features, have already been known relatively well for decades (for a review, see Ref. [41]), e.g., the amount of DNA offered must be sufficient. Although quantities of 0.1 ng/mL of recipient cell suspension could already elicit an effect, good results are often only obtained at concentrations around 100 ng/mL. On average, 1 out of 100 treated bacterial cells becomes transformed. Double-stranded DNA is suitable for transformation, with a minimum size corresponding to about one gene. Single-stranded DNA can only be taken up in special cases.

The starting point for transformation experiments is the extraction of the DNA to be used from the bacterial donor strain. For this purpose, the cells are usually broken down mechanically in combination with detergent treatment, the protein is extracted by phenol/chloroform, and the nucleic acids of the supernatant are precipitated with ethanol. After resuspension in saline, the DNA is freed from RNA, which is also present, by RNase treatment and then cleaned further if necessary. This DNA preparation of a donor strain is then added to a well-growing culture of the acceptor strain. After just a few minutes, DNA uptake is completed as revealed by subsequent DNase treatment of the cells, which will no longer interfere.

When extracted from bacteria, the DNA breaks down into a larger number of fragments, due to shear forces that normally occur during processing. The genome of *B. subtilis*, with an approximate molecular weight of 10^9^, thus yields DNA pieces with an average molecular weight of 25 × 10^6^. Each of these pieces of DNA contains only about 1/40 of the genes present in the whole genome. The number of genes per fragment can be estimated at about 100 to 150. There is only a chance of transforming an acceptor cell if it receives a piece of DNA with the necessary gene or gene part. The probability of this is low when considering a unique piece. However, it is improved by the fact that a large number of such DNA pieces can penetrate many bacteria per culture.

In addition to the form and amount of DNA offered, the physiological state of the bacterial cells used as acceptors is also important for the success of the transformation. It is said that under optimal conditions, all “competent” cells become transformed. The proportion of competent cells in the total population changes with the growth conditions. Competence refers to a state in which the cells tend to take up DNA and allow further processes leading to successful transformation. Competence often only reaches its maximum towards the end of the exponential growth phase and then drops just as quickly. The molecular basis of competence originates on the one side from changes in the structure of the cell wall, which make it permeable to large molecules, and on the other side from the synthesis of certain receptors exposed on the bacterial surface that specifically bind double-stranded DNA molecules of a certain minimum size.

This binding can take place if the cell has been activated by so-called competence factors and if an energy source, e.g., sugar, is available. The binding makes the DNA insensitive to DNase. Their temporal course can therefore be followed by DNase treatment of cells at different intervals after the addition of the DNA and monitoring of the respective transformant numbers. Binding is followed by the actual uptake of the DNA into the cells through the cell wall and PM. In *Pneumococci* and *B. subtilis*, the DNA must be offered as a double strand, but only single-stranded DNA arrives in the recipient cells. It was shown that one of the two strands is degraded during absorption. Corresponding oligonucleotide fragments are released outside the cells. In *H. influenzae*, the DNA was supposed to enter the acceptor cells as a double strand. Early data on the uptake of DNA in the transformation of *Pneumococci* have shown that Mg^2+^ or Mn^2+^ ions, but also a nuclease, are involved. If this nuclease were genetically defective, the uptake no longer took place, but the DNA was still bound to the outside of the cell wall.

It was assumed that the energy gained from the degradation of one strand by the corresponding nuclease serves to absorb the other strand, i.e., the nuclease may act as a “translocase”. On the other hand, a mutant was identified in which the binding of DNA to the cells is no longer possible. This mutant demonstrated the necessity of prior binding for the subsequent uptake of DNA.

The process of transformation in Gram-positive bacteria, e.g., *B. subtilis* [42], can be divided into the following stages: (i) development of competence due to secretion of a small protein called competence factor; (ii) binding of double-stranded DNA molecules; (iii) uptake of single-stranded DNA molecules; (iv) coating of single-stranded molecules with a specific protein that protects DNA from nucleases (eclipse complex); (v) integration by recombination of the transformed DNA strand into the acceptor chromosome; (vi) replication of integrated DNA segments and segregation of acceptor and donor alleles in progeny bacteria to yield transformed clones with new phenotypes.

In Gram-negative bacteria, e.g., *H. influenzae* [43], no competence factor appears to be produced, and the process of DNA binding and uptake differs from that of Gram-positives. Thus, only homologous DNA is specifically bound, and DNA is taken up in the form of intact double-stranded molecules. Unlike the species described above, *Escherichia coli* cannot be transformed effectively without special treatments, i.e., competence must be induced by appropriate experimental procedures. This may be accomplished by removing most of the cell wall to produce spheroplasts [44] or alternatively, the cell envelope can be rendered permeable to DNA by treatment with Ca^2+^ ions and subjection to heat shock [45]. The latter method, originally developed for the transfection of *E. coli* by lambda DNA, in which the transformed cells do not survive, was subsequently shown to be effective for the transformation of bacteria with plasmid DNA [46], in which transformed cells are required to survive the treatment. Transformation of large plasmids (>30 kb) is not very efficient, and the uptake of chromosomal DNA is limited to strains carrying specific mutations in their recombination systems (recB^−^, recC^−^, sbc^−^), which lack exonuclease V and are unable to degrade incoming linear DNA fragments [47,48].

#### 2.2.2. Mechanisms of Lower Eukaryotes

The initial situation for the transformation of lower eukaryotes was different from that of bacteria, since the genetic material of eukaryotes, apart from certain stages of cell division, is organized into nuclei into which the donor DNA, if it is offered from the outside of the cell, must first penetrate for its appropriate incorporation into the genome of this cell. The nuclear membrane apparently impedes or even prevents this penetration.

If the donor DNA does not reach the cell nuclei and is not incorporated into the eukaryotic genome, there is still the possibility that this DNA will establish itself in the cytoplasm as an additional genetic material, function there, and replicate. Then, there would not be transformation per se, but rather transfection. This refers to the infectious transfer of naked DNA genomes, e.g., from bacteriophages, to acceptor cells without incorporating foreign DNA into their genome, but with its replication. Today, similar cases are called transformation, where ring-shaped plasmid DNA penetrates bacterial cells, replicates there independently of the bacterial chromosome, and expresses the genetic information it contains.

Characteristic of the uncertainty in the field of transformation in eukaryotes were the first reports [49], which were much cited at that time. According to them, the injection of erythrocytic nuclear complex or DNA from whole blood of ducks/guinea hens of one variety/race into ducklings/chickens of another variety/race gave rise to animals with an altered phenotype. The findings could not be reproduced by other working groups or by the authors themselves [50]. Experiments with many other animal objects as well as with simpler eukaryotes, such as yeast or the mold *Neurospora crassa*, initially yielded only negative or doubtful results (for a review, see Ref. [51]). It was only in the following years that some work became known, the results of which looked more positive.

In the case of simple eukaryotes, a series of 25 yeast strains was tested. They belonged to the genera *Saccharomyces*, *Hansenula*, and *Candida*. Members of the same genus as well as different genera, in pairwise combinations, served as DNA donors and acceptors, respectively [51]. Physiological characteristics were the degradability of nine different sugars and the ability to synthesize adenine. Of the 109 combinations examined, 11 yielded clear indications of transformation. Under optimal conditions, some combinations gave up to 14% transformants. A weakness of the experiments was that the yeast strains used, apart from the adenine mutants, were only phenotypical, but not genetically characterized [52].

Subsequent work with *N. crassa* yielded far fewer transformants, but the mutants used here were genetically very precisely characterized and therefore allowed a detailed genetic analysis of the transformants obtained. The acceptor was mainly an inositol-requiring mutant, in the form of a mycelium, and the wild type served as the donor. DNA was extracted from it, and RNA was extracted in other experiments [53,54,55]. Here, as in analogous experiments on bacterial transformation, only those cells grew into colonies that were themselves capable of synthesizing inositol. Such colonies were actually received, subsequently isolated, bred, and finally backcrossed with the inositol-needing acceptor strain. The values obtained with DNA (387 prototrophic cells in 405 × 10^6^ cells tested, corresponding to a rate of 0.95 × 10^−6^ compared to 0.04 × 10^−6^ when using saline solution) were disadvantaged by the fact that by no means all experiments yielded prototrophic cells, but only 37 out of 55 [54,55].

If one surveys what has been said about transformation in eukaryotes till midst of the 1970s, it can be said that even the more recent experiments did not yet provide a uniform picture [56]. If the phenotypically modified cells of a eukaryote treated with DNA are called transformants, without at the same time assuming a genotypic change comparable to that of bacterial transformants, it can be said that the transformation rates of unicellular eukaryotes are generally considerably lower than those of bacteria. It seems to be well documented that not all eukaryotes tested so far, and also not all mutants or mutations of a particular tested object, can receive transformation-like changes in the course of the addition of pure DNA (for a review, see Ref. [57]). Even with bacteria, transformation is by no means always possible.

In conclusion, all the above findings, raised in the course of the next three decades following Griffith’s transformation experiment, were based on and confirm the DNA-centric view of inheritance. DNA was thought to be both necessary and sufficient to provoke phenotypic changes, i.e., small features and minor differences, at least in acceptor bacteria. Thus, the focus on the replication and transfer of solely DNA concomitantly led to disinterest for analogous processes ensuing the biogenesis of (sub)cellular structures, such as biological membranes and organelles, as well as the maintenance of metabolic fluxes, such as regulatory (feedback) loops and turnover of cellular constituents and metabolites (for further discussion, see below and Ref. [16]). This resulted in extreme reductionism with ultimately narrowing of cellular heredity down to DNA heredity in a sequential fashion with exclusion of cybernetic, topological, and macromolecular heredity under accompanying sequential elimination of non-DNA matter, i.e., regulatory, structural, and self-replicating matter as well as information. The delineation of the agency of the various (f)actors contributing to this biological reductionism, including its putative consequences with regard to the creation of novel microorganisms, in general, and lower eukaryotes, in particular, as well as with regard to limitation of the predictability, controllability, and manipulability to solely small features and minor differences rather than total building plans and large differences represents the topic of this review.

## 3. Putative Experimental Designs for the Creation of Novel Microorganisms

### 3.1. Designing Novel Bacteria

I would like to suggest a design for a Griffith transformation experiment 2.0 that relies on the inheritance of both DNA and non-DNA matter, capable of replicating and transferring both “structural” and “regulatory” matter and information, thereby enabling topological, cybernetic and ultimately cellular heredity.

#### 3.1.1. Early Conceptions on Horizontal Gene Transfer

With the exception of a few researchers, among them Theobald Smith [58,59], nobody in the last decades of the 19th century till the first half of the 20th century was aware of a bacterial culture typically representing a population of distinct, disparate cells. Consequently, Louis Pasteur spent little—technical or intellectual—effort in generating pure cultures [60]. Even Robert Koch, the most critical competitor of Pasteur, interpreted most findings on the variability within bacterial species to be caused by contamination. Together with F. Cohn, he insisted on the hypothesis of the “monomorphism, i.e., the genetic stability or invariance of all bacterial species (for a review, see Ref. [61]. With the initiation of genetic studies in the field of bacteriology, the first research on the variability of virulence factors in cultures of aging bacteria was conducted by Werner Braun [62,63]. He gave a rather simple explanation in terms of “rough variants” apparently spontaneously emerging in those cultures, which may exhibit certain selective advantages. The speculation about “symbiotic particles”, which can be transferred within a bacterial culture and were subsequently termed plasmids, was born rather early and found to be very attractive for being involved in bacterial inheritance [64]. This caused Joshua Lederberg to subsequently study the effect of loss of those plasmids as a possible explanation for Pasteur’s expression of “attenuation” [65,66]. And in fact, cultures of non-virulent *Bacillus anthracis* were later found to be devoid of a plasmid mediating virulence (for a review, see Refs. [67,68]). In the course of “pasteurization,” plasmids are removed from cultures of virulent bacteria, thereby confirming their critical function in the production of toxin crystals, e.g., in *Bacillus thuringiensis*. During the second half of the 20th century, the features of virulence and toxicity were repeatedly found to be transmissible within a given bacterial species [69,70,71] and then, in the course of their culturing [72], demonstrated to be based on plasmids [73,74].

In addition to the above line of “monomorphism” and its refutation by the expression of “symbiotic particles”, an alleged polymorphism of bacteria was reported, a phenomenon called “cyclogeny” [75,76]. Variants of the old idea of pleomorphism were widespread between 1916 and 1935 [77,78]. But “cyclogeny” was not a return to the extreme views about the existence of only one species of bacteria, but it contradicted speculation about the supposed “simplicity” of the bacterial life cycle (monomorphism). At that time, it had already been proven many times that bacteria have a complexity of their life cycle that is quite comparable to that of “higher fungi” [78]. In the 1920s, there were further reports that different types of bacteria could pass through a submicroscopic stage, which was so small that these types of bacteria could pass through typical bacterial filters and were subsequently able to reproduce the original cell type from which they had been derived [79]. Stanford bacteriologist W.H. Manwaring commented in 1934 [80]: “*About the only conventional law of genetics and organic evolution that is not definitely challenged by current bacteriologists is the nineteenth century denial of the possibility of spontaneous generation of a bacterial cell. Even this is questioned by certain recent theories in their hypothetical transformation of certain normal enzymes into ‘pathogenic genes’ or ‘filterable viruses’, and in their apparent success of ‘Twort genes’ by the chemical oxidation of certain heat-sterilized organic products…..Whether or not future refinements in immune-chemical technique can or will bridge the gap between the apparent Lamarckian world of bacteriology and the presumptive Darwinian world of higher biological science is beyond current prophecy*”.

The fact that bacterial species per se can go through certain stages of development successively was based on the claim that several physiological, serological, morphological, and behavioral traits can vary in combination. Later, in the 1940s, researchers succeeded in demonstrating that the bacterial characteristics of a culture are capable of independent variation and cannot be regenerated by sub-microscopic filterable particles [81]. However, the fact that bacteria reproduce exclusively asexually or parasexually by fission was still the most common assumption. In 1939, the British cytogeneticist C.D. Darlington referred to bacteria as being asexual, lacking gene recombination, and still being undifferentiated from viruses [82]. The great English evolutionary biologist, philosopher, eugenicist, and writer Julian Huxley summarized in 1942 [83] what “*everyone knew about bacteria: Bacteria, in spite of occasional reports of a sexual cycle, appear to be not only wholly asexual but pre-mitotic. Their hereditary constitution is not differentiated into specialized parts with different functions. They have no genes in the sense of accurately quantized portions of hereditary substances, and therefore, they have no need for accurate division of the genetic system, which is accomplished by mitosis. The entire organism appears to function as soma and germplasm, and evolution must be a matter of alteration in the reaction system as a whole. That occasional ‘mutations’ occur we know, but there is no ground for supposing that they are similar in nature to those of higher organisms, nor, since they are usually reversible according to conditions, that they play the same part in evolution. We must, in fact, expect that the processes of variation and evolution in bacteria are quite different from the corresponding processes in multicellular organisms. But their secret has not yet been unraveled*”. As Joshua Lederberg noted in one of his first review articles on microbial genetics in 1946 [84]: “*The lack of outward differentiation of bacteria and viruses does give the appearance of holo-cellular propagation and identity between direct transmission and inheritance. Geneticists and bacteriologists alike have shown justifiable hesitation in accepting unanalyzed genetic variations as gene mutations*”.

In 1927 Frederick Griffith [85] and then in 1946 Joshua Lederberg together with his collaborator Edward Tatum [86,87], opened up a new field of bacterial genetics when they experimentally studied *S. pneumoniae* and *E. coli*, respectively, and demonstrated parasexual processes or–to call it scientifically–genetic recombination (for a review, see Refs. [88,89]. Studies of genetic mutations and mechanisms of genetic regulation in bacteria, conducted by Jacob and Monod in the 1950s, suggested that the earlier Lamarckian interpretations were partly due to the fact that reversible physiological adaptations (i.e., induced enzyme formation) had been mixed up with more or less irreversible hereditary “mutations” (for a review, see Ref. [90]). Subsequently, bacterial geneticists demonstrated that bacteria possess various inheritance mechanisms that were not known to be effective in plants and animals. In addition to gene transfer from parental/mother to offspring/daughter cells, i.e., vertical gene transfer, there is also transfer of genes between different evolutionary lineages, i.e., horizontal/lateral gene transfer (HGT/LGT). A bacterium of a certain strain may have acquired one or more genes from a completely foreign strain or organism. Therefore, similarities and differences in some genes may not be a measure of genealogical relatedness (for a review, see Ref. [91]). For example, if organism type A and organism type B possess the same gene for a protein, it may not be because they both belong to the same taxonomic group, but because one of them acquired that gene through transformation from a third type of organism that is not descended from them. HGT/LGT could, in principle, blur genetic ancestry.

Since the mid-1950s, it has been known that bacteria have several mechanisms for transferring genes between unrelated groups–through transformation, conjugation (chromosome transfer), and transduction (mediated by bacteriophages). However, the importance of these mechanisms in bacterial evolution has mostly been overlooked [92]. Bacterial geneticists tend to look at bacterial mutations and gene exchange between related strains. In the late 1940s, when the cause of bacterial antibiotic resistance was discussed among bacteriologists, discussions revolved around the question of whether acquired resistance was the result of environmentally induced adaptive and then inherited changes or of mutations and natural selection. HGT as the cause of widespread antibiotic resistance was not considered until a decade later, when Japanese researchers argued for it. In 1960, they reported that antibiotic resistance can be easily transmitted between *E. coli* and *Shigella* in the gut of humans [93]. This year, studies on mixed cultures showed that cell-to-cell contact or conjugation was essential for this. Acquired antibiotic resistance thus represented what Lederberg had called “*infectious heredity*”.

Towards the end of the twentieth century, HGT between bacteria was recognized as an urgent medical problem in relation to antibiotic resistance. At the same time, analyses of complete genome sequences led bacterial evolutionary theorists to suspect that the extent of HGT was far greater than previously thought. The importance of HGT for bacterial phylogeny was not a completely new topic in genomic studies. This was found at the beginning of the era of a new molecular morphology for bacterial phylogenetics. Roger Stanier had already recognized this problem in 1971 when he wrote the following [94]: “*Genetic studies on procaryote are complicated by a phenomenon not known to exist among eucaryote. A bacterium may be a genetic chimera, some of its phenotypic traits being determined by episomes that are transferable among (and expressed in) a considerable range of species, having markedly different chromosomal genomes. It is therefore conceivable that false inferences concerning the relatedness of a series of bacteria could be reached by the study of one or more shared characters determined by episomal genes*”.

#### 3.1.2. Paragglutination

During the years 1890 and 1930, it was heavily discussed whether certain features, such as surface antigens, were capable of being transferred from certain donor to acceptor bacteria within a given culture or environment [95]. As a possible mechanism underlying this putative phenomenon, it was frequently speculated that it relied on the “absorption of heterologous receptors”, as manifested in the appearance of sensitization of collodion particles, erythrocytes, or bacteria in a passive fashion. In 1909, it was reported that cells of *E. coli* originating from a case of dysentery were able to react with antiserum against *Shigella flexneri*, which maintained its cross-reactivity with dysentery *E. coli* cells subsequently isolated from that patient. The possibility of transfer of antigenic features from *Shigella* to *E. coli* cultures of these bacterial species were grown together. In fact, the latter acquired cross-reactivity from the former. An analogous finding with regard to *E. coli* and *Salmonella paratyphi* was subsequently reported by Eugene and Elisabeth Wollman in 1925 [96]. They related the putative transfer of surface antigens or receptors to the bacteriophage phenomenon and introduced the terms “paraheredity” and “parasexuality”.

However, in the course of retrospection, experiments in vitro for the demonstration of paragglutination should be discriminated from the exceptional appearance of cross-reactivities in natural environments, e.g., strains of *E. coli* displaying almost complete antigens which originated from *Salmonella* or *Shigella*. No doubt, the latter type of acquisition of surface antigens or receptors is certainly operative in nature and may be interpreted as a typical case of genetic recombination following either transformation and chromosome transfer by competent bacteria, or transduction by lysogenic phages. Moreover, a number of microorganisms express a widely distributed rough antigen, causing cross-reactivity of certain bacterial strains, which consequently become classified as (R) variants. In the course of culturing those variants in the laboratory, the selected antigen-specific bacteriocides may encompass complement, colicins, and phages and could have been transferred between bacteria by one of the above molecular mechanisms prior to genetic recombination rather than by direct transfer of receptors. However, this conception has faced considerable criticism and doubts. Nevertheless, for reasons preliminarily addressed in a previous study [16] and discussed in depth in the present manuscript, the latter phenomenon may be explained by the above — still viable and sound — hypothesis, which deserves its experimental confirmation or refutation in the future.

#### 3.1.3. Current Concepts on HGT

It was generally thought that HGT affects (metabolic) genes for accessory functions, but not genes in the “heart” of the organism, and especially those whose products interact with many proteins. However, after 1965, with the advent of bacterial genomics, a significantly higher incidence of HGT cases has been detected [97]. In 1998, Howard Ochmann and Jeffrey Lawrence reported that about 10% of the *E. coli* genome is made up of genes that were likely acquired in more than 200 HGT events following the divergence of *E. coli* and *Salmonella* about 100 million years ago [98]. Accordingly, about 18% of the *E. coli* genome has been acquired relatively recently [99]. These data suggested that HGT may have a profound impact on evolutionary comparisons of genomes [100].

Consequently, HGT blurs the lines between “species” [101]. The ease with which genes are exchanged between bacteria reinforced the long-held view that the concept of biological species (in the general sense of a reproductively isolated group) does not apply to bacteria [102]. Isolation mechanisms that segregate Mendelian populations do not apply, and testing for sterility of the hybrids is irrelevant. Certainly, many bacteriologists of the 1950s and 1960s had already recognized that the concept of species was not applicable to bacteria. They did not assume this because bacteria could exchange genes between distantly related groups, but because laboratory studies suggested that sexual reproduction was a rare event for bacteria, as it was for most microorganisms [103]. Samuel T. Cowan concluded in 1962 [104] that “*the microbial species does not exist; it is impossible to define except in terms of nomenclatural type; and it is one of the greatest myths of microbiology*”.

Before the advent of bacterial genomics, there was little thought about the importance of HGT, although speculation about its ubiquity had led some theorists, such as Sorin Sonea, to conceive of the entire bacterial world as a single “super-organism” [105,106]. Speculation about the nature and intensity of HGT led some bacterial phylogeneticists at the end of the Millennium to fear that the entire enterprise of classification was obsolete and that a natural phylogeny of bacteria was impossible. Other phylogeneticists remained convinced of molecular phylogeny. Radhey Gupta, based on studies of various protein phylogenies, argued that the bacterial world has a fundamental dichotomy between what he called “monoderm” (bacteria that have a single membrane) and “diderms” (those with a double membrane), corresponding to Gram-positive and Gram-negative bacteria, respectively [107].

In the meantime, Carl Woese reconsidered an idea he had begun to develop already in 1982 [108,109,110]. He interpreted the new genomic evidence, which suggested that the “archaea”, the “bacteria”, and the “eukarya” have many genes in common, in relation to intense HGT that occurred between the groups before they turned out to be different fundamental domains. The evidence could be understood in terms of its long-sought transitional stages in the evolution of the translational apparatus. Ever since Darwin, biologists have assumed that all life on Earth emerged from a single ancestral cell. Thus, Darwin had argued that [111] “*probably all organic beings which have ever lived on this earth have descended from one primordial form, into which life was first breathed*”. But Woese contradicted the canon of a single ancestral mother for all cells. He speculated that instead of the expected “first cell,” the ancestor was a population of pro-cellular entities with underdeveloped and error-prone replication and translation machineries. Before the development of the modern replication and translational apparatuses, evolution was driven by a different mode and pace. At this early stage, there were no individual lineages that could be distinguished as such due to the many gene mutations and intense HGT. These processes would very quickly have generated enormous diversity. Primitive systems were supposed to be modular and able to exchange parts freely, but with the further development of the replication and translation machineries, which had become limiting for HGT, definable lineages also changed. This represented the great Darwinian split from which the three domains of archaea, bacteria, and eukaryotes emerged from the chaos.

Woese suggested the emergence of the three domains as follows [112]: There would first be a period of intense genetic “heating” (i.e., high mutation rates and intense gene transfer between lineages that would have a short history) in which the cellular units would have remained simple and the information systems would have operated inaccurately. It would have remained impossible to recognize organismic genealogies in it. This intense period was followed by a genetic “cooling”, whereby the development of the modern cell with sophisticated replication and translational apparatuses led to the emergence of genealogically recognized domains and taxa. So Woese came to the following conclusion [108,109,110,112]: “*The universal ancestor is not a discrete entity. It is, rather, a diverse community of cells that survives and evolves as a biological unit. This communal ancestor has a physical history but not a genealogical one. Over time, this ancestor refined into a smaller number of increasingly complex cell types, with the ancestors of the three primary groupings of organisms arising as a result*”.

In the mid-1990s, the U.S. Department of Energy launched a Microbial Genome Initiative, which served as an offshoot of the Human Genome Project and was initiated five years earlier together with the National Institute of Health. In 1995, researchers at the Institute for Genomic Research (TIGR), led by J. Craig Venter, published the sequence of *H. influenzae* [113]. The following year, Woese and Olsen, together with researchers from the TIGR, published the complete sequence of the first archaebacterium, *Methanococcus jannaschi* [110]. The Human Genome Project had been considered useful in terms of its medical use, and microbial genomics was justified in a similar way—each microbe for a specific practical purpose, i.e., medical, agricultural, or industrial [110,112,113].

As early as 1996, the complete sequence of the methanogenic bacterium *M. janaschii* revealed that its genome consists of certain groups of genes that are much more similar to eukaryotic than bacterial ones, while other groups of genes are much more closely related to their bacterial homologues [109]. Eugen Koonin and coworkers confirmed that the *M. jannaschii* genes for translation, transcription, replication, and protein secretion are more similar to eukaryotes than bacteria [114]. The authors interpreted this to mean that methanogenic bacteria are composed of eukaryotes and eubacteria. Using rigorous phylogenetic methods, the presence of two superclasses of genes in prokaryotes that have different relationships to eukaryotic genes has been discovered. In studies of the genomes of *E. coli*, *Syneccocystis* PCC6803 (a cyanobacterium), *M. jannaschii*, and *Saccharomyces cerevisiae*, it was found that *M. jannaschii*’s information genes, which code for gene products responsible for processes such as translation and transcription, are most closely related to those in eukaryotes [115,116,117,118]. However, the operational genes of eukaryotes, which are responsible for the normal function of the cell (so-called “housekeeping genes”), are most closely related to their counterparts in *E. coli* and *Syneccocystis* [118]. This provided the definitive proof that the eukaryotic cell is a chimera of eubacteria and members of the methanogenic bacteria or eocytes (an eocyte genome was not available at the time).

HGT is also widespread in eubacteria. For example, it was detected in *A. aeolicus*, although little consistency could be found in the reconstruction of the relationships between a number of operational genes [119]. Comparative analyses of *E. coli* open reading frames (ORFs) revealed that 675 ORFs are most similar to *Synechocystis* PCC6803 [115], 231 ORFs to *M. jannaschii* [109], and 254 ORFs to *S. cerevisiae* [116]. Using a degenerated base composition and codon use as a measure of a foreign gene, Ochman, Lawrence, and colleagues [99,120] argued that 755 out of 4288 *E. coli* ORFs have been acquired from *Salmonella* in 234 events since *E. coli* divergence from Salmonella about 100 million years ago.

Although HGT is widely used, it does not happen by chance. In an insightful phylogenetic analysis, the frequency of HGT in operational genes was assessed in the following six prokaryotic proteomes, namely *E. coli*, *Synechocystis* PCC6803, *B. subtilis*, *A. aeolicus*, *M. jannaschii*, and *A. fulgidus* [117,119,121,122]. To prove kinship, orthologues of these six prokaryotic genomes were examined to determine the extent of HGT. All tests showed very clearly that operational genes have been continuously transferred horizontally between prokaryotes since the appearance of the last common ancestor of life, i.e., the hypothetical “primordial cell”. To explain why operational genes are more likely than informational genes to induce HGT, the so-called “complexity hypothesis” has been proposed. This postulates that informational genes are not as likely or efficient to support HGT because they are often components of large complexes. In contrast, however, the products encoded by operational genes are usually not part of large complexes and are therefore more easily transmitted [122].

It is becoming increasingly clear that HGT has had a major impact on the evolution of life on Earth. It is considered a major agent, perhaps the main agent, that has been and still is responsible for the spread of genetic diversity by transferring genes across species boundaries [123]. By rapidly introducing newly developed genes into existing genomes, HGT bypasses the slow step of ab initio gene formation, thereby accelerating genome innovation. An analysis of about 20,000 genes expressed in eight free-living prokaryotic genomes showed that HGT is preferentially found in organisms that share similar parameters in terms of environmental influences and genomic factors. These include genome size, genome G/C composition, carbon utilization, and tolerance to oxygen [124] (for a review, see Ref. [125]). Based on the number of prokaryotic species within the exchange communities, this study concluded that HGT accelerated the introduction of new genes into new species by a factor of 10,000. Indeed, HGT may have been, or still is, responsible for a remarkable increase in genome innovation that surpasses anything that could have been achieved by clonal evolution alone.

#### 3.1.4. Current Conceptions on Mechanism and Specificity of Transformation

As already discussed, transformation is a widespread mechanism of HGT in bacteria (for a review, see Refs. [126,127]). In Gram-negative bacteria uptake of DNA into the periplasmic compartment requires a pilus structure specific for a given DNA and the DNA-binding protein ComEA. The DNA is first transported through the OM by retraction of the pilus for subsequent final and irreversible interaction with periplasmic ComEA, acting as a “Brownian ratchet” to prevent backward diffusion. A related molecular mechanism probably also operates in Gram-positive bacteria, although the corresponding systems are much less well characterized so far. Transport, defined as the movement of a single strand of transforming DNA into the cytosol, depends in Gram-positive and negative bacteria on the function of the channel protein ComEC, which is probably driven by proton symport.

Most bacteria are able to take up DNA without any requirement or preference for specific sequences. A few bacterial species, such as *N. gonorrhoeae* and *H. influenzae*, require that the transforming DNA molecules have the so-called DNA uptake signal sequences DUS (ATGCCGTCTGAA) or USS (AAGTGCGGT plus two short T-rich segments) [128] for maximal transformation efficiency. USS determines the affinity for the minor DU pilus protein ComP. This limitation of DNA uptake in these two and a few related bacterial species may be explained by the fact that their constitutive competence, i.e., the ability to be transformed by foreign DNA (see above), requires an effective defense mechanism against invading foreign DNA.

The development of competence is mediated by the synthesis and release of the so-called competence factor CSP [129,130]. This small peptide of 41 amino acids with similarity to bacteriocins is a translation product of the genes comC1 and comC2 [131,132,133]. It enables the induction of the expression of genes encoding transformation-relevant proteins upon its processing, i.e., cleavage of the amino-terminal signal sequence of 24 amino acids, to the active versions, CSP-1 and CSP-2. In non-competent bacterial cultures as well as in many encapsulated bacterial strains, the addition of CSP-1 or CSP-2 can trigger competence [131], accompanied by differential regulation of the expression of more than 100 genes [134,135,136,137]. The induction of competence by CSP leads to the release of transformable DNA from competent cells [138,139,140,141]. This release depends crucially on the expression of the major autolysine LytA and the autolytic lysozyme LytC.

The natural transformation of *S. pneumoniae* can be divided into several steps: (i) induction of competence; (ii) binding of the double-stranded DNA at the cell surface; (iii) degradation of the double-stranded DNA into discrete double-stranded fragments; (iv) uptake of single-stranded linearized DNA fragments; (v) binding of the internalized DNA to single strand-specific binding proteins; and (vi) homologous recombination of the linear single-stranded foreign DNA into the host chromosome by a single strand-specific displacement mechanism.

Essentially, any DNA can be taken up by *S. pneumoniae*, as in most cases (for exceptions see above), no specific recognition and signal sequences seem to be required. The majority of DNA, which is not homologous to pneumococcal DNA or fails to undergo independent replication, is degraded and thus flows into the pool of nucleotide precursors. The degradation of the DNA into relatively small fragments does not exclude the possibility of uptake and integration of large DNA fragments into the host chromosome, since carefully prepared DNA fragments may mediate the transformation of genetic markers, that are more than several 100 kilobases apart. Occasionally, a foreign DNA, that is not homologous to the host chromosome but is a physically coupled component of a homologous DNA, can be integrated into the host chromosome via a molecular mechanism, which has been described as homology-driven illegitimate recombination.

In order to survive under various stress-related environmental conditions, bacteria have developed a system to acquire possible beneficial extrinsic genes from neighboring strains via natural transformation (for a review, see Ref. [127]). The natural transformation mechanisms of Gram-negative and Gram-positive bacteria have now been well studied (for a review, see Ref. [126]). In Gram-negative bacteria, foreign DNA can be transported via type IV pili, which consist of PilA, PilB, PilM, PilN, PilO, PilP, PilQ, PilT, PilF, TsaP, and FimV proteins (for a review, see Ref. [142]) or, in Gram-positive bacteria, via pseudopili [143]. Subsequently, a single strand of foreign DNA becomes degraded in the periplasmic compartment, and parallel to this, the other single strand is released into the cytoplasm via the transmembrane channel composed of ComEC proteins. Most ComEC proteins have an amino-terminal soluble domain, which is probably responsible for the transport process of the DNA single strand [144], as well as a carboxy-terminal domain, which is responsible for the degradation of the non-transformable DNA strand [145]. In the cytoplasm, the DNA-processing protein A (DprA) is then used to integrate the single-stranded foreign DNA into the host chromosome in a RecA-dependent manner [146].

For some bacteria, specific physiological conditions are required to achieve competence. In most transformable bacteria, the natural transformation takes place under specific growth conditions, which also holds true for *S. pneumoniae* [147]. In contrast, in a few bacterial species, such as *N. gonorrhoeae* (for a review, see Ref. [148]), their natural transformation takes place in all active growth phases and is thus referred to as “constitutively competent”. In addition, it has been shown that antibiotic stress or DNA damage can lead to the induction of transformation in some bacteria, such as *S. pneumoniae* [149]. Moreover, for some bacterial species their transformation competence is stimulated by antibiotics [150]. On the other hand, bacteria have developed specific defense mechanisms against natural transformation to reduce the uptake of potentially harmful foreign DNA. For example, in some of the naturally competent bacteria, specific DNA sequences are required for DNA uptake by host or acceptor cells, such as *N. gonorrhoeae* [151] (see above), and some host cells produce nucleases to degrade foreign DNA. In addition, in bacteria, certain immune systems, such as the system of restriction and modification, the CRISPR-Cas system [152], and the Agos system [153], play an important role in limiting natural transformation. Thus, bacteria are equipped with both natural transformation apparatuses and defense systems to face the constantly changing environmental conditions.

It has been reported that *N. gonorrhoeae* DNA interacts with the minor pilin component ComP of their type IV pili, which exhibits extraordinary binding specificity for DUS, and thus a function of ComP as a receptor for DUS and in the initiation of the DNA uptake [154]. In other bacterial species, sequence-specific receptors have not yet been identified, and most appear to take up DNA without any preferences regarding the type of foreign DNA. One possibility is that these bacterial species have alternative mechanisms to resist foreign DNA that are not realized in *Neisseriaceae*.

#### 3.1.5. Micelle-like LP/LPS Complexes as Non-DNA Matter of Inheritance in Bacteria

On basis of the outcome of the Griffith transformation experiment, as performed according to design 1.0 and 1.1 (see Figure 1 and Figure 2 and Table 1), it was not possible to clarify whether passage of *Pneumococci* through the host organism, i.e., mice, is necessary to induce the production of any matter of inheritance as a consequence of specific (incubation) conditions prevalent only in living systems. With the successful introduction of cell-free systems (design 1.2 and 1.3; see Table 1), this question seemed to be adequately addressed. Whole-cell homogenates, subcellular fractions, or water-soluble (chloroform) extracts without or with subsequent heat treatment prepared from (S) *Pneumococci* turned out to manage the transformation of (R) into (S) *Pneumococci* of the same serotype. This apparently demonstrated that passage through the host organism, as well as the use of intact donor bacteria, is not a prerequisite for bacterial transformation. Nevertheless, the possibility that the generation of non-DNA matter of inheritance, such as PM vesicles and micelle-like LP/LPS complexes, critically depends on hitherto unknown (catalytic or stoichiometric) activities intrinsic to total host organisms and/or dependent on intact donor bacteria cannot be excluded so far.

It may be of relevance that studies with rat adipocytes and human erythroleukemia cells have revealed the operation of non-DNA matter of inheritance in cultured mammalian cells [155,156,157,158] (for a review, see Refs. [159,160]). It was shown that under certain environmental conditions, such as high glucose, oxidative stress, radicals, mechanical pressure, subsets of glycosylphosphatidylinositol-anchored proteins (GPI-AP; [161], for a review, see Refs. [162,163,164,165]) are released from the outer leaflet of the donor or mother cell PM into micelle-like complexes together with (lyso)phospholipids, cholesterol, cytoskeletal elements and transmembrane proteins into the culture medium [166,167]. Transfer of those micelle-like GPI-AP complexes to acceptor cells and incorporation into their PM led to upregulation of lipid and glycogen synthesis, respectively [155,156,157,158]. Importantly, those apparently acquired phenotypes or traits were stably maintained for the next generations of acceptor or daughter cells. The micelle-like GPI-AP complexes transferred may form specific structures, such as protuberances, blebs or invaginations, and be replicated by a concerted action ranging mechanistically between the principles of self-assembly and self-organization (autopoiesis) and amenable to topological re-arrangement in response to certain environmental cues, rather than solely by self-assembly.

This raises the intriguing questions as to whether micelle-like LP/LPS complexes are actually expressed in bacteria and may operate as counterparts of micelle-like GPI-AP complexes in mammals, thereby operating in a similar fashion during replication, intercellular transfer, and expression of novel features in the acceptor bacteria. GPI-AP are modified by a carboxy-terminal GPI anchor, which is identical from yeast to man and consists of amphiphilic phosphatidylinositol (PI) as the phospholipid component and a highly conserved hydrophilic glycan core (for a review, see Refs. [162,163,165]. It consists of a non-acetylated glucosamine and three mannose residues connected via specific glycosidic linkages. One end is glycosidically linked to PI, and the other non-reducing end is invariably amide-linked to the carboxy-terminus of the protein moiety via a phosphoethanolamine diester bridge. Typically, the phospholipid component is built from a PI moiety with two long-chain (mostly saturated) fatty acyl chains, as either diacyl-PI or 1-alkyl-2-acyl-PI.

**Figure 3:**
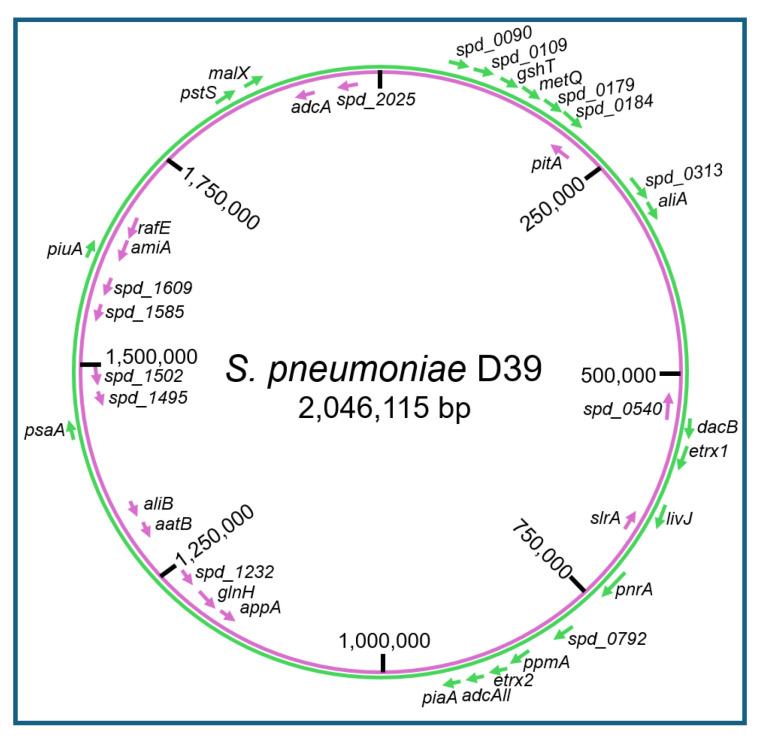
Genes for the biogenesis of LP in *S. pneumoniae*. Map of the genes engaged in the biogenesis of LP with their localization, distribution (annotation according to the common gene database of bacteria) and orientation (green arrows represent the coding strand, pink arrows the reverse strand) along the chromosome as depicted in a circular configuration of the pneumococcal genome (not drawn at scale, according to Refs. [22,23,168]).

**Figure 4:**
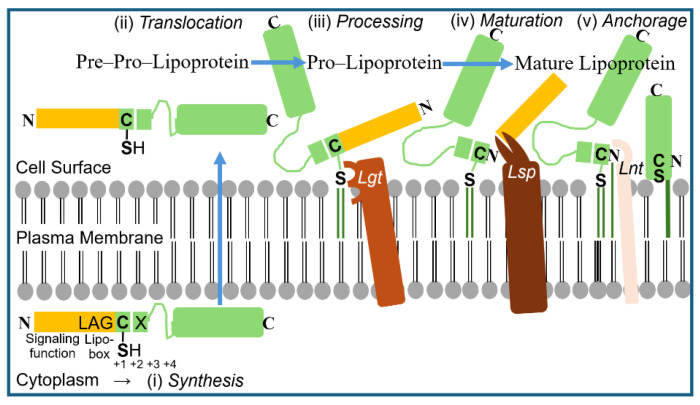
Biogenesis of LP in *S. pneumoniae*. The blue arrows indicate the translocation of the preprolipoprotein synthesized in the cytoplasm across the PM, followed by processing to pro-lipoproteins and final maturation to mature lipoprotein under concomitant anchorage at the outer leaflet of the PM (cell surface) (see text for details).

Thus, eukaryotic GPI-AP and prokaryotic LP share the amphiphilic overall nature, with the protein and carbohydrate moieties displaying the hydrophilic and the esterified fatty acyl residue(s), the hydrophobic portions (Figure 3 and Figure 4).

The biogenesis of LP in Gram-positive bacteria proceeds along four distinct stages. (i) LP are synthesized as higher molecular-weight pre-pro-LP precursors in the cytoplasm, which are equipped with bipartite amino-terminal signal peptides mediating translocation across the PM (signaling function) and subsequent processing and maturation (characteristic consensus sequence with lipobox motif). (ii) Pre-pro-LP are translocated along the tat or sec pathway into the immediate surface area of the PM. (iii) Here, pre-pro-LP become processed by the covalent addition of a diacylglyceryl moiety to the thiol group of the highly conserved cysteine within the lipobox motif (lipidation) with the aid of the enzyme LP diacyl glyceryl transferase (Lgt). (iv) Subsequent to this processing, pro-LP is converted to mature LP by removal of the signal peptide, including the lipobox, with the aid of the enzyme LP signal peptidase II (Lsp). (v) In some, but not all Gram-positive bacteria (among them *S. pneumoniae*), the newly generated amino-terminal cysteine residue of the mature LP, already harboring the diacyl glyceryl moiety, is modified by the addition of a fatty acyl moiety to its previously free amino terminus via an amide linkage with the aid of the enzyme LP N-acyl transferase (Lnt). Thus, this version of a mature LP is stably anchored at the PM on the basis of three covalently attached fatty acyl residues. Furthermore, the correct localization of mature LP at the PM is also determined by the nature of the amino acids at positions +2 to +4 (Figure 4).

**Figure 5:**
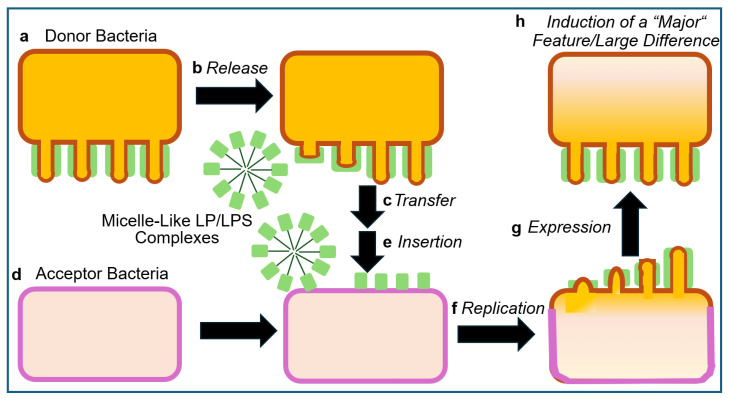
Hypothetical model for the transformation of acceptor bacteria by micelle-like LP/LPS complexes for the induction of a major feature or large phenotypic difference. (**a**) Donor bacteria display a considerable number of “membrane landscapes” of complex configuration, e.g., protuberances, which are assembled by interactions between (glyco)phospholipids, LPS, lipidated (i.e., double or triple fatty acylated) LP (indicated in green as the surface covering the protuberances) and peripheral as well as transmembrane elements of the bacterial cytoskeleton (not indicated here). (**b**) Micelle-like LP/LPS complexes constituted by the same components (only the lipidated LP as green cuboids with the acyl chains indicated as single green lines are depicted under omission of all other putative micellar components) become released from the “membrane landscapes”, e.g., protuberances. (**c**) Upon transfer of the micelle-like LP/LPS complexes to acceptor bacteria (**d**) which lack protuberances, (**e**) the components of the complexes, among them the lipidated LP, become inserted into the acceptor PM. (**f**) Replication of the “membrane landscapes”, which are configured around the newly inserted components of the transferred complexes, prior to the next cycle of release, may be explained by a hypothetical molecular mechanism, called PM memory. (**g**) This and the ongoing DNA-dependent expression of lipidated LP and other components of the complexes (**h**) leads to transformation of the acceptor bacteria exhibiting a major feature or large difference in the course of the gradual expansion of the “membrane landscapes”, e.g., protuberances, over their surface. The gradual increase in intensity from light to dark brown color along steps (**g**) and (**h**) indicates the continual phenotypic chance from acceptor to donor bacteria.

This similarity of the amphiphilic configuration between mammalian GPI-AP and bacterial LP prompted the speculation that subsets of the latter may also be arranged in non-covalently assembled micelle-like complexes in the nm-scale together with other transmembrane proteins, cytoskeletal elements, LPS, and other (lyso)/(glyco)phospholipids. These putative micelle-like LP/LPS complexes may resemble in structure the LP particles required for signaling and “hitch-hiking” between mammalian cells [166,167]. They could serve as non-DNA matter of inheritance in bacteria, capable of replication, transfer and induction of novel features and phenotypes, and be configurated in the PM as nanodomains or so-called “membrane landscapes” of specific topology, such as protuberances, blebs and invaginations (Figure 5).

As a first hint in this direction, it has previously been reported that certain bacterial LP are secreted across the PM into the periplasm and beyond [169].

#### 3.1.6. “Blending” of Bacteria

**Figure 6:**
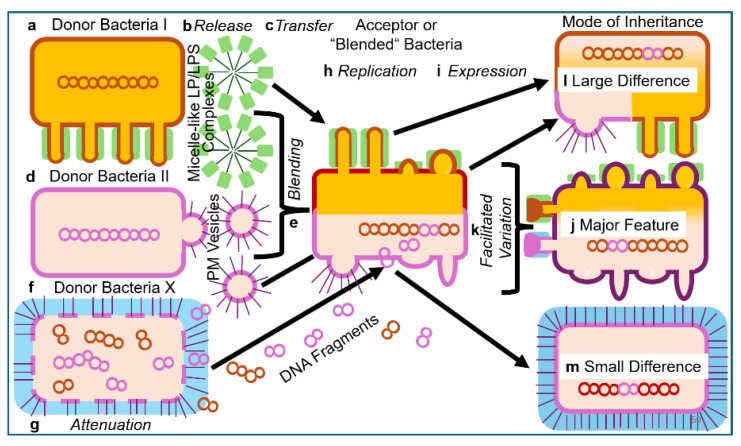
Hypothetical model for the transformation of bacteria by “blending” of various matters and structures of inheritance. (**a**) Donor bacteria I display a considerable number of protuberances of a given configuration at their PM. Micelle-like LP/LPS complexes which harbor the components specific of “membrane landscapes”, such as protuberances, are released from donor bacteria I into the surrounding medium, (**b**) subsequently transferred to acceptor bacteria (**c**) which lack those “membrane landscapes” of the same type, i.e., protuberances, and finally inserted into PM of acceptor bacteria. (**d**) Donor bacteria II exhibit a few “membrane landscapes” of a given structure (indicated as thin pink lines) at their PM which become released as PM vesicles (**b**) and then (**c**) transfer their constituent components (transmembrane, peripheral, lipidated, cytoplasmic, cytoskeletal protein, LP, LPS) to PM of acceptor bacteria for final fusion. (**e**) Donor bacteria I and II mutually exchange or mix parts/portions of their PM and cytoplasmic contents to varying degrees, including the associated architecture and configuration, substance pools, metabolite fluxes, and morphogenetic gradients, and their mutual interactions through regulatory circuits. This resulted in structurally altered “membrane landscapes”, e.g., protuberances, with intermingling of both materiality and structure as well as recombination of DNA molecules. (**f**) Donor bacteria X release DNA fragments in the course of attenuation (**g**), which are then taken up by acceptor bacteria for subsequent recombination into their genome. (**h**) Replication of the “membrane landscapes” proceeds as a consequence of transfer and insertion or fusion of the “feet”, i.e., micelle-like LP/LPS complexes or PM vesicles, and subsequent leaving behind of “footprints” (see below for a hypothetical mechanism). (**i**) The ongoing expression of “membrane landscapes” (i.e., the “footprints”) and micelle-like LP/LPS complexes (i.e., the “feet”) of a given configuration in concert with protein expression directed by the recombined genome leads to transformation. (**j**) The acceptor bacteria will exhibit a novel major feature, displayed by neither donor bacteria I nor II. (**k**) This process may be further supported by so-called “facilitated variation”, further fostering mutual intermingling of substance, shape, and substance and regulatory fluxes between donor bacteria I and II. In contrast, replication, release, transfer, and expression of non-DNA and DNA matter or, alternatively, solely DNA mediates the inheritance of large (**l**) or only small differences (**m**), respectively.

The “blending” or “mixing” of (Gram-positive as well as Gram-negative) bacteria presumably represents a very rare or even impossible event in natural environments due to impairment of cell fusion by the PM/OM and/or cell wall. However, culturing at high cell density and under specific experimental conditions, e.g., enzymic digestion of the cell wall, incubation with Ca^2+^ or polyethylene glycol (PEG), may foster “blending”, “mixing” or fusion of two different bacterial donor strains resulting in acceptor bacteria, which exhibit features originating from both donors at varying degree. These “hybrid” bacteria displaying mixed phenotypes of novel quality may be identified using appropriate selection criteria (e.g., for altered morphology and/or physiology) coupled to automated high-throughput analysis (e.g., of images or metabolism; see Table 1). Depending on the extent of “blending”, large differences and major features in comparison to the two bacterial donor strains may be generated, which ultimately are the result of the formation of mixtures or “hybrids” between structural and regulatory information and matter (Figure 6).

Alternatively, or in addition, micelle-like LP/LPS-complexes or PM/OM vesicles may transfer components of “membrane landscapes”, such as protuberances or blebs, from donor to acceptor bacteria. Certainly, the “blending” process becomes supported by the parallel exchange of DNA fragments between the donor bacteria in the course of recombination and subsequent initiation of the synthesis of novel components required for hybrid “membrane landscapes” in the acceptor or “blended” bacteria. In contrast, attenuation of donor bacteria by heat treatment (design 1.1) will cause transformation of acceptor bacteria solely by the released DNA fragments, leading to inheritance of only small differences (Figure 6).

#### 3.1.7. Bacterial Cytoskeleton

By no means, bacteria represent simple “bags” of enzymes but rather are equipped with complex intracellular structures which extend and challenge the current understanding of the operation and function of the cytoskeleton in eukaryotic cells (for a review, see Refs. [170,171]. The bacterial cytoskeleton is formed by a multitude of cytoplasmic protein filaments and their super-arrangements, such as rods, rings, twisted pairs, tubes, sheets, spirals, moving patches, meshes, and composites thereof. They facilitate the movement or scaffolding, i.e., stabilization, positioning, and recruitment, of other intracellular materials, according to the specific functional needs of each super-arrangement. The term “skeleton” has been introduced to characterize the material basis or support of an object. The term cytoskeleton has been adapted to explain the emergence and function of a network of long and thin structures as operational in the cytoplasm of eukaryotic cells, where it determines their shape. Those thin structures were later identified to consist of tubulin, actin, and intermediate filament proteins. They may cause the movement of objects via the induction of their own assembly and disassembly, operate as fixed tracks for motor proteins, and serve as linkers and scaffolding factors in order to provoke the positioning and stabilization of other cellular materials. The subsequent identification of bacterial homologs of tubulins, actins, intermediate filament proteins, FtsZ, MreB, and crescentin, elicited the–at first unexpected–knowledge that also bacteria harbor a cytoskeleton responsible for their shaping and subcellular organization/compartmentalization. Albeit the bacterial filaments have also been recognized to trigger the pulling and pushing of intracellular structures as well as connecting them together as linkers and scaffolding factors, a function as tracks for bacterial motor proteins has remained unknown so far. However, the nature of the bacterial cytoskeleton is critically affected by both the small size of the cell, which necessitates a format of filament bundles being nearly as long as they are wide (and thereby not corresponding to typical filamentous structures), and by the various novel non-tubulin, non-actin, and non-intermediate filament super-arrangements (i.e., assemblies of higher structure). According to literature (for a review, see Ref. [172]), these elements display a multitude of characteristics of the typical eukaryotic cytoskeleton. Meanwhile, MreB, FtsZ, and crescentin have been reported to operate as bacterial members of large families of diverse homologs of eukaryotic cytoskeletal proteins. In addition, bacterial cytoskeletal proteins apparently lacking eukaryotic homologs, such as WACA proteins and the deviant Walker A-type ATPases bactofilins, have been identified; moreover, a number of universally conserved polypeptides, such as the metabolic enzyme CtpS, are known to provoke their assembly into filamentous structures. They may catalyze the biogenesis of cytoskeletal structures and their functions and ultimately be responsible for the evolution of the bacterial cytoskeleton from self-assembling enzymes (for a review, see Ref. [173]).

Recently, with the aid of in vivo multidimensional fluorescence microscopy in combination with modern structural imaging techniques, such as cryo-electron tomography supported by advanced light microscopy, the dynamics and remarkable plasticity of the (intra)cellular structures and architecture of bacteria were visualized (for a review, see Ref. [174]). In conclusion, in bacterial cells, the assembly of various proteinaceous components into dynamic cytoskeletal filaments which fulfill multiple essential functions, such as growth, morphogenesis, cell division, DNA partitioning, and cell motility, in addition to shaping and structuring the cell, is now well established. Albeit the majority of the filaments manage to assemble on their own to provoke structures of higher order, there is increasing evidence that bacteria express a number of polypeptides which undergo polymerization only with the aid of a matrix, such as DNA, prion proteins, micelle-like LP/LPS-complexes, PM and OM, or other (polymeric filamentous or prolonged flat) structures. Those matrix-assisted polymeric (filamentous) super-arrangements and families of filament-forming systems often heavily depend on the hydrolysis of nucleotides and are strongly cytomotive. They must therefore be regarded as a vital part of the cytoskeleton in both prokaryotes and eukaryotes, which are engaged in vital cellular processes encompassing DNA and chromosome segregation, DNA maintenance and repair, gene silencing, mitosis, and cytokinesis (for a review, see Ref. [175]).

**Figure 7:**
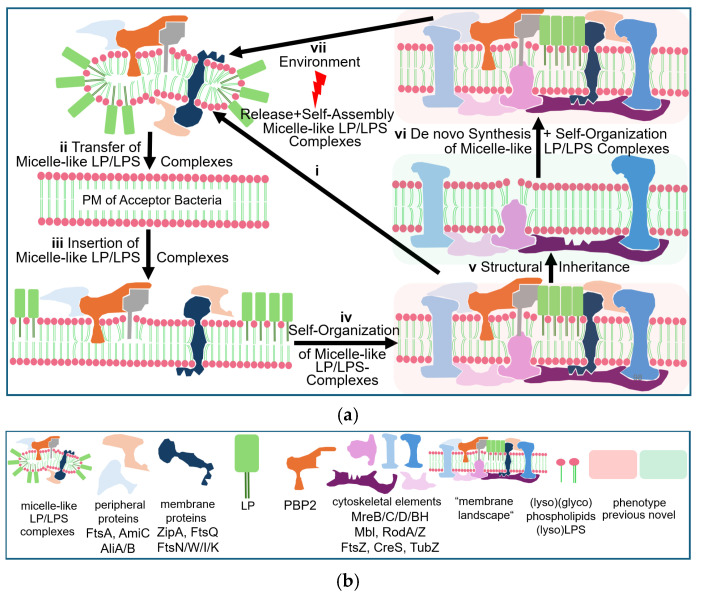
(**a**) Hypothetical model of the transfer of micelle-like LP/LPS complexes and the replication of “membrane landscapes” by operation of the so-called “PM memory”. (i) Micelle-like LP/LPS complexes consist of lipidated (i.e., double or triple acylated) LP, transmembrane and peripheral membrane proteins as parts of the bacterial cytoskeleton and (lyso-)(glyco)phospholipids, among them LPS, and are presumably formed by self-assembly. They are released from (Gram-positive) donor bacteria either spontaneously or in response to certain environmental stimuli. The latter may affect the specific configuration and topology of the complexes when associated with PM of the donor cells or upon their release into the surroundings. (ii) Micelle-like LP/LPS complexes are subsequently transferred to the PM of acceptor bacteria. (iii) Micelle-like LP/LPS complexes are (spontaneously) inserted into certain “membrane landscapes” of the PM of acceptor bacteria at specific sites. These are formed by pre-existing (cytoskeletal) protein components at the PM in conjunction with those pre-existing at the surface layer immediately below their cytoplasmic face. The inserted micelle-like LP/LPS complexes cause the rearrangement of lipidic and proteinaceous cytoskeletal components and thereby trigger the self-organization of “membrane landscapes” of identical molecular composition and topology. (iv) As a consequence, micelle-like LP/LPS complexes can be regarded as a “three-dimensional foot” which, upon insertion into acceptor PM, leaves behind a “footprint”. These are formed by cytoskeletal elements in concert with (lyso-)(glyco)phospholipids and LPS in the course of self-organization. It is assumed that the interaction between “footprint” and “foot” blocks the activity of (newly) arranged “membrane landscapes” with regard to phenotypic consequences, leading to self- or autoinhibition of the former by the latter. Thereby, the putative insults of the inheritance of (small or large) differences or novel (minor or major) features are masked, and the “old” phenotype (as indicated in weak red) is maintained as a result of the transferred micelle-like LP/LPS complexes. (v) Dissociation of the micelle-like LP/LPS complexes, i.e., the feet, reliefs the “membrane landscapes”, i.e., the “footprints”, from self- or autoinhibition, causing emergence of a “new” phenotype (as indicated in weak green). In conclusion, this process may exemplify structural inheritance/templating which is mediated by micelle-like LP/LPS-complexes and enables the continuous biogenesis of “membrane landscapes” as well as on-off switching of the phenotype, in parallel. (vi) The biogenesis of micelle-like LP/LPS complexes from de novo synthesized components relies on self-organization and thereby guarantees maintenance of the autoinhibited state of the “membrane landscapes” (as indicated in weak red). Completion of their synthesis and the increasingly improved fit between “foot” and “footprint” is linked to “dilution” with time of the inherited novel phenotype in favor of the previous one. (vii) Micelle-like LP/LPS complexes are prepared to initiate the next cycle of inheritance of non-DNA matter in the course of their release from the “membrane landscapes” into the culture medium or natural surroundings upon exposure to environmental stimuli. Dissociation relieves “membrane landscapes,” i.e., the “footprints”, from self- or autoinhibition, causing switching of the previous to the novel phenotype. (**b**) Legend for the above model.

The formation of “membrane landscapes” in the course of self-organization with pre-existing components of cytoskeletal elements (see below Figure 7a, steps iv and vi), as well as through self-assembly with de novo synthesized components of micelle-like LP/LPS complexes (see below Figure 7a, step i), may rely on weak linkages and, in consequence, enable “facilitated variation”.

By analogy, the “PM memory” presented in this model may resemble the “water memory”, which has recently been proposed as a molecular mechanism for the homeopathic principle [176,177,178,179,180,181]. According to the “PM memory”, micelle-like LP/LPS complexes may operate in analogy to drug molecules; the (glyco)phospholipids of the PM of acceptor bacteria with incorporated pre-existing cytoskeletal elements may operate in analogy to water molecules (Figure 7).

The “PM memory” has previously been suggested as the replication mechanism for micelle-like GPI-AP complexes in mammalian cells [24]. As already mentioned, the expression of a cytoskeletal architecture, which represents a prerequisite for the above hypothetical model, has long been known in eukaryotic cells and has meanwhile been amply documented for bacteria, too (for a review, see Refs. [170,171,172,173,174,175]).

#### 3.1.8. Ranking of Various Experimental Designs of “Blending” and Transformation According to Their Putative Potential of Exclusion and Bias

The various experimental designs envisaged for “in vitro transformation” and “in vivo blending” and classified according to the source of the transforming principle, which had already been performed in the past (X) or remain to be carried out, were compared with regard to the experimental configuration and detection method, with calculation of the exclusion index (E) in declining order from DNA to cells as well as of the bias strength (B) in declining order from capsule serotype to virulence to morphology/physiology (Table 2).

The sum T of the numeric values of E and B was calculated for each experimental system and detection method, leading to a relative ranking R each. Accordingly, in vivo “blending” in intact mice of acceptor bacteria by co-injection of donor bacteria was ranked at R1–3, followed by whole-cell homogenate at R2–4, then non-DNA matter, such as PM vesicles and micelle-like LP/LPS complexes at R3–5, and finally purified DNA at R5–6, with monitoring of morphology and physiology being least biased with B1, followed by that of virulence with B2 and then of the serotype with B3. This relative ranking held also true for in vitro transformation of acceptor bacteria upon direct incubation with donor bacteria (R4–5), followed by that of whole-cell homogenate (R4–6), then non-DNA matter (R5–7) and finally purified DNA (R6–8), with monitoring of morphology and physiology being less biased than that of serotype.

### 3.2. Designing Novel Eukaryotic Unicellular Microorganisms

#### 3.2.1. Somatic Cell Nuclear Transfer

The first experiments on the reprogramming of somatic cells have historical roots that go far back into the history of developmental biology. As early as 1888, the German zoologist August Weismann postulated that differentiation determinants are unevenly distributed among the daughter cells during embryonic development and that this process of (asymmetrical) cell division enables cells to differentiate into different cell types [182]. Of course, this was accompanied by the assumption that daughter cells are no longer equivalent to each other in terms of genetic information. Weismann concluded that during development, part of the genetic information is lost in the daughter cells [183]. In other words, during cellular differentiation, cells lose genetic information and are therefore no longer genetically equivalent to each other.

This theory of mosaic development was confirmed by initial experiments performed by Wilhelm Roux. He killed one cell of frog embryos of the two-cell stage with a hot needle and subsequently obtained only half-embryos from the other surviving cell [184]. He concluded that already in the two-cell stage, the two daughter cells are no longer equivalent to each other. The first doubts about Weismann’s theory were raised by the work of the developmental biologist Hans Driesch, who carried out similar experiments on sea urchin embryos [185]. He divided sea urchin embryos of the two-cell stage into two completely separate cells and observed how a complete sea urchin larva grew from each of the two isolated cells. His conclusion from these experiments was that in the two-cell stage, the two cells are still genetically equivalent [185]. Conversely, this also means that Weismann’s theory, if it is valid at all, can only apply to a later stage of development.

**Figure 8:**
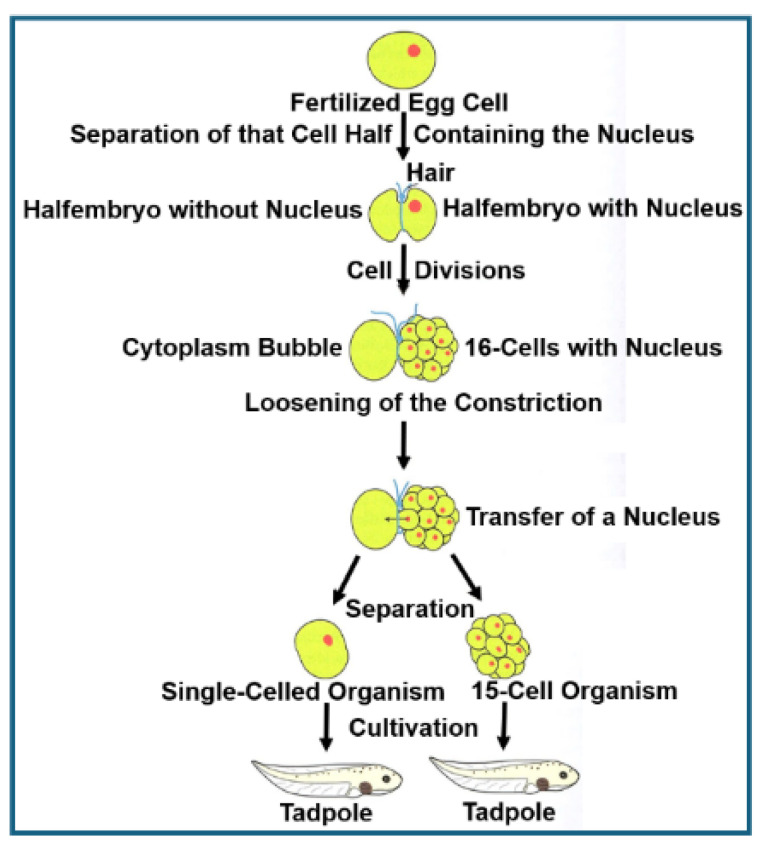
Experiment on the genetic equivalence of cell nuclei in the early embryo. Spemann used early salamander embryos, whose fertilized eggs he separated in half with a fine hair, generating a half-embryo containing the nucleus and a half-embryo consisting only of cytoplasm [187]. The half-embryo with genetic information now began to divide further and generate new cells. When the embryo part that was active in division was in the 16-cell stage, Spemann loosened the loop so that a cell nucleus could pass into the cytoplasmic bubble of the second half-embryo. He then completely laced the two half-embryos into two separate structures with the help of the hair. This resulted in an embryo formed by 15 cells and at the same time one embryo consisting of only a single cell, which, however, contained the nucleus of a 16-cell embryo. After cultivation, both embryos developed into normal free-floating tadpoles.

At first glance, the observations of Roux and Driesch were, of course, contradictory. It should be noted that in his experiments, Roux did not completely separate the killed cell from the other surviving cell with the hot needle. Rather, the remains of the dead cell were still in contact with the surviving cell. Today, we know that a complete separation of the killed cell would very well have led to the formation of an embryo from the surviving cell. This evidence was later provided by the German zoologist Hans Spemann [186]. In his experiments, he used thin baby hairs formed as a snare to separate the two cells of a frog embryo in the two-cell stage. He was able to observe that one complete embryo was created from each of the two cells [187]. Subsequent experiments were also able to prove the equivalence of the genetic information (even) at a later stage of development. For example, the German-American biologist and experimental physiologist Jacques Loeb investigated the development of sea urchins that were stimulated to divide by parthenogenetic reproduction (for a review, see Ref. [188]). Loeb stimulated the cell division of an egg cell in the absence of fertilization by changing the milieu of the surrounding water. For this purpose, he only introduced MgCl_2_ into the solution at higher concentrations. In a few cases, however, this increase in intracellular ion concentration was so great that the developing embryos burst upon their re-exposure to water. In this process, membrane vesicles were formed by chance that were filled with cytoplasm and at the same time contained a cell nucleus. Surprisingly, a sea urchin larva also developed from these artificial structures, so that Loeb concluded that the cell nucleus must still contain all the information for the development of a complete living being at a later point in time (for a review, see Ref. [188]). Hans Spemann undertook a similar experiment with salamander embryos (for a review, see Ref. [186]). Again, he used a thin hair, this time for the separation of a fertilized egg, and thereby generated a half-embryo containing the nucleus and a half-embryo containing only cytoplasm (Figure 8).

Using this experimental protocol, Spemann was thus able to show that all cell nuclei are genetically equivalent up to the early morula stage and that no genes are lost during cellular differentiation until this point in development [189]. In addition, Spemann introduced a new experimental concept. By the transfer of the cell nucleus into the previously constricted cytoplasmic bubble, the cell nucleus was brought into a different cytoplasmic environment. If it were in the cytoplasm of an early embryo with 16 cells before the transfer, it was brought back into an environment typical of the fertilized egg cell by the transfer. In 1938, Spemann then formulated the daring experiment in 1938 that the nucleus of a differentiated cell would have to be transplanted into an enucleated egg cell in order to refute Weismann’s hypothesis. This experiment proposed by Hans Spemann, which seemed very futuristic in the 30s of the last century, was first implemented two decades later.

In the 1950s, a number of scientists continued to investigate the question of a possible loss of genetic information during embryonic development with the help of nuclear transplantation experiments. The English zoologists Robert W. Briggs and Thomas J. King finally succeeded in the transplantation of nuclei from the frog *Rana ripiens* into a previously enucleated egg cell of the same species [190]. To remove the nucleus from the egg, they used a thin cannula for sucking out the nucleus. This process is also known as aspiration. Also, the subsequent insertion of a “new” cell nucleus into this enucleated egg cell was performed with a microcannula. This process is also known as nuclear transfer. The experiment showed that the embryos reconstituted in this way only developed into normal embryos if the nuclei originated from early blastula embryos. If, on the other hand, cell nuclei were removed from endodermal tissue of later tadpole stages and inserted into the enucleated egg cells, the experiment failed. Briggs and King concluded that during the development from late blastula to tadpole, irreversible changes take place in the genome, leading to the elimination of genes required for early development [191]. In doing so, they initially supported Weismann’s theory.

**Figure 9:**
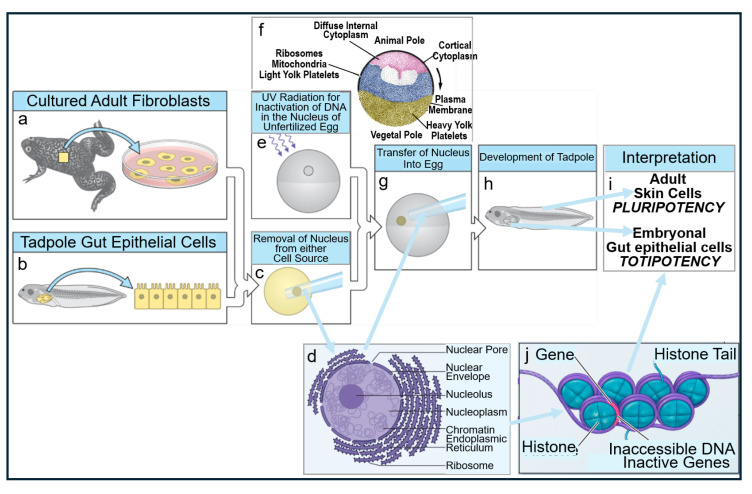
Canonical interpretation of nucleus transplantation experiments. (**a**) Small pieces excised from the skin of the frog *Xenopus leavis* were transferred to culture dishes and cultured into fibroblasts (blue arrow). (**b**) Intestinal epithelial cells (enterocytes) were prepared from the gut of tadpoles of *X. leavis* (blue arrow). (**c**) Nuclei were isolated from the fibroblasts or enterocytes by aspiration. (**d**) Those nuclei harbor chromatin, in addition to other structures, such as the nuclear envelope, an extension of the endoplasmic reticulum equipped with ribosomes (blue arrow). (**e**) Egg cells were removed from another animal, and their nuclei were inactivated by UV radiation. (**f**) Those egg cells exhibit a complex structure encompassing the animal pole, cortical and diffuse internal cytoplasm, light and heavy yolk platelets, and the vegetal pole, with unequal distribution of “Weismann’s differentiation determinants”. (**g**) The nuclei of either source were transplanted into the enucleated egg cells (primordial or mature oocytes) by microinjection (blue arrow). (**h**) In both cases, viable tadpoles developed (blue arrows). (**i**) Tadpoles either failed to develop into adult frogs (donor nuclei derived from adult skin cells) or succeeded in the completion of differentiation (donor nuclei derived from tadpole enterocytes). This difference in differentiation potential or “potency” has been interpreted as (limited) “pluripotency” and (almost unlimited) “totipotency”, respectively. (**j**) According to the canonical view, the chromatin, which is constituted by DNA wrapped around histone proteins (blue arrow) and regulates gene expression in the course of its post-translational modification, primarily determines the developmental fate of the embryonic (totipotent) or adult (pluripotent) organism (blue arrow) through (epigenetic) control of the accessibility of DNA, with a secondary role of the egg cytoplasm, only.

At the beginning of the 1960s, Sir John Gurdon tackled this question again using the South African clawed frog *Xenopus laevis* and transplanted cell nuclei obtained from intestinal cells of an adult tadpole [192]. In the end, he succeeded in generating adult female and male frogs that were identical to the donor. This represented the starting point for the generation of clones from animals. With this experimental approach, Gurdon was able to prove for the first time that DNA from differentiated cells is able to support embryonic development at the level of the egg cell (Figure 9).

Apparently, the question of a change in genetic information during development was thus clearly answered: Even differentiated cells still possess the complete genetic information necessary for the course of embryonic development. However, these experiments also indicated that there are most likely factors in the egg cell that ensure that the nucleus of an already differentiated cell is reprogrammed. In his experiments, Gurdon also used for the first time a genetic marker to prove beyond doubt that the newly developed organism carries the genetic material of the donor cell nucleus. He made use of a specific *Xenopus* mutant, one-nucleolate (1-nu), which produces only one nucleolus in each cell, while normal wildtype animals have two nucleoli each [193]. By analyzing the cell nuclei after their transfer, Gurdon was able to show with this mutant that the developing organism contains the genetic material of the donor embryo, exclusively (for a review, see Ref. [194]).

It should be noted here that it has not yet been possible to transplant a cell nucleus obtained from the tissue of an adult frog (e.g., fibroblasts or enterocytes), rather than a tadpole, into enucleated egg cells and thereby to generate an adult frog in turn (see Figure 9i, upper section). Due to the relatively fast cell cycle of early *Xenopus* embryos, with cell division occurring approximately every 20 min, the time that the genetic material is in contact with the reprogramming factors, before differentiation is initiated, is probably too short [195,196]. An alternative interpretation argues for the critical role of cytoplasmic factors in the egg cell, which obviously control the donor genome and cause the actual reprogramming. Epigenetic markers characteristic of the differentiated state may be removed, and those typical of the fertilized zygote or embryonic stem cells are introduced (see Figure 9j). Here, it is easy to imagine that this mechanism is all the more successful the longer the cytoplasmic factors have time to act on the genome.

Weismann’s hypothesis of unevenly distributed differentiation determinants in daughter cells, Roux’s and Driesch’s experimental elimination of one cell of the two-cell stage of frog and sea urchin embryos, respectively, Loeb’s hypoosmotic shock-induced formation of membrane vesicles which are filled with both cytoplasm and a cell nucleus, Spemann’s experiment with separating fertilized eggs of early salamander embryos in half with a fine hair (see Figure 8) and Brigg’s, King’s and Gurdon’s nucleus transplantation experiments (see Figure 9) have two important implications in common (for a review, see Refs. [197,198]): (i) The daughter cells prior to development into the blastula stage, the one cell of the embryonal two-cell stage, the hypoosmotic membrane vesicles equipped with both cytoplasm and nucleus, and the donor nuclei transferred into acceptor egg cells they all possess specific structural (e.g., endoplasmic reticulum, ribosomes, nuclear envelope, PM) as well as cybernetic (e.g., regulatory loops, metabolite and energy fluxes, reprogramming factors) matter and information. Consequently, the “pluri-/totipotency” or “agency” of the nucleus can neither be attributed to only its DNA matter being causally sufficient nor only to the egg cell operating as an empty water-filled bag with the potential for self-assembly of its internal structures under the direction of the egg cell DNA. Rather, the apparent “agency” of the nucleus critically depends on the concerted action of the total matter and information inherited from parental to daughter cells or between mitotic cells and thereby guaranteeing cellular heredity. (ii) The observation that a tadpole or even a mature frog can develop from an egg cell upon microinjection of intact donor DNA of any nature, e.g., from either tadpole fibroblasts or mature frog enterocytes, strongly argues for the “potency” or “agency” of the egg cell rather than that of the transferred donor nucleus or DNA. Apparently, the egg cell is capable of using the “foreign” nucleus or DNA as information for the synthesis of new polypeptides and nothing else, according to its needs for the initiation, maintenance, and determination of an organism’s development under the sole control of the non-DNA matter and information of the egg cell. Thus, egg cells seem to order proteins both in quantitative and qualitative respects, just as a typical customer orders goods from a catalog of a commercial supplier.

If one wants to use an anthrophomorphic vocabulary, then the behavior of egg cells or mitotic cells could be described as “egoistic”, “parasitic”, or “selfish”. This view represents a reversal of Richard Dawkin’s conception of sociobiology, in which cells and organisms are sometimes construed as little more than the playthings of self-replicating genes manipulating whole cells and organisms in order to promote and propagate their own “selfishness” [199]. However, in most reviews and textbooks, the terms “pluri-/toti-/omnipotency” are used for the demonstration of the agency of the DNA or nucleus to solely determine the fate of the cell. This canonical DNA- or nucleus-centric view has limited our potential in creating novel microorganisms (see below). It should be replaced by an alternative interpretation reconsidering macromolecular, structural/topological and regulatory/cybernetic matter and information, which, in concert with the DNA/nucleus, is required to support cellular heredity and development.

#### 3.2.2. Transfer of Macromolecular, Structural, and Regulatory Matter and Information for Synthetic Biology

**Figure 10:**
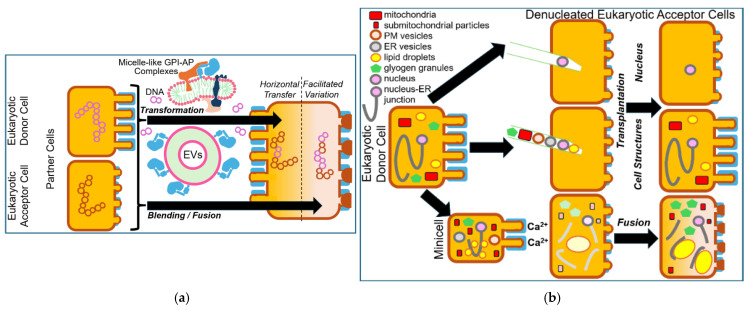
Hypothetical model for the creation of eukaryotic unicellular cells relying on various strategies. (**a**) Horizontal transfer of micelle-like GPI-AP complexes and EV together with DNA fragments from donor to acceptor cells in the course of transformation or “facilitated variation” (see below for further details) of differing portions of partner cells in the course of their “blending” or fusion. This leads to (re-)configuration of various materials as well as structural (in vitro transformation and in vivo “blending”) and cybernetic (“blending”) information, and is accompanied by the generation of novel protists. Those will display completely altered phenotypes compared to the partner cells and reflect major differences compared to both donor and acceptor cells. (**b**) Transplantation using the method of microinjection of intact nuclei alone, which are constituted by nucleoproteins and nuclear membranes, with the latter being formed as an extension of the endoplasmic reticulum and derived from eukaryotic donor cells, or of a mixture of nuclei, PM and ER vesicles, lipid droplets, mitochondria, glycogen granules, cytoplasmic contents, etc. Either of these materials is transplanted into eukaryotic acceptor cells, which have been enucleated by irradiation with UV light. Consequently, latter cells will develop novel phenotypes, such as protuberances of pronounced length, typical of the PM from the donor cells (middle section). In contrast, transplantation of nuclei alone will not result in the emergence of acceptor cells displaying novel features, such as PM protuberances of pronounced rather than short length (upper section). Fusion of mini- or protocells displaying all the typical features of eukaryotic donor cells, including PM protuberances of pronounced length, with enucleated acceptor cells will putatively lead to the emergence of “hybrid cells” of completely novel phenotype with characteristics resulting from the transferred and mixed materials of both donor and acceptor cells, such as PM protuberances of intermediate length and mixed protein composition.

In the following, hypothetical strategies for the use of DNA/nuclear, macromolecular, structural, and regulatory information in concert for the creation of novel unicellular eukaryotes are presented, which are based on micelle-like GPI-AP complexes, extracellular vesicles, and minicells (Figure 10). 

Micelle-like GPI-AP complexes (for a review, see Refs. [157,158,159,160,161,162,163,164,165,166,167]) and EV (for a review, see Refs. [200,201,202,203,204,205,206,207]) are constituted by GPI-AP embedded together with other membrane proteins, (lyso)phospholipids, and cholesterol in micelle-like structures and small vesicles, respectively. These two basic entities enable the transfer of GPI-AP from mammalian donor to acceptor cells, thereby initiating stable phenotypic changes in the latter [154,155]. Mini- or protocells are derived from eukaryotic donor cells and can be generated by exposure to hyper/hypoosmotic shock.

The results obtained are explained best by a selection of the protein and, consequently, gene expression by the enucleated acceptor cells according to their needs. This emphasizes the “passive” role of the donor cell DNA in making available the information for the synthesis of proteins, exclusively, as a “catalog for ordering of proteins by cells”. In contrast, the transfer of non-nuclear structures, such as the endoplasmic reticulum (microsomes), PM vesicles, mitochondria, lipid droplets and glycogen granules, together with the transplantation of intact nuclei, which in concert represent the so-called “unit membranes and structures”, could provide sufficient information for both the assembly of the cellular architecture and the synthesis of proteins. Transplantation and transfer in concert may lead to the expression of novel features, such as protuberances of the PM, typical of the donor cells.

It seems worth mentioning that lipid droplets as well as glycogen granules could be regarded as non-DNA matter of inheritance, too. Upon their dispersion into smaller elements in the acceptor cells and subsequent transfer to the donor cells, they could serve as a sort of priming molecules for the addition of neutral triacylglycerol moieties and glucose units to the growing/expanding lipid droplets and nascent glycogen granules, respectively. This mechanism of replication, transfer, and phenotypic consequences exerted by lipid droplets and glycogen granules (for a review, see Ref. [208]) resembles a putative priming function of LPS S(III). This LPS necessitated considerable efforts in excluding its involvement as a non-DNA matter of inheritance in *Pneumococci* [209]. Moreover, recent experimental findings hint at a role of lipid droplets in the transfer of a subset of GPI-AP from large to small adipose cells within mammalian tissues, which argues for the inheritance of the capacity to synthesize and store lipids from the former to the latter [208,210].

Finally, the fusion of mini- or protocells derived from eukaryotic donor cells [211,212] with enucleated acceptor cells may trigger the (re-)configuration of various materials as well as the structural and cybernetic information. This should be accompanied by the emergence of acceptor cells, which display totally novel phenotypes compared to the donor cells, such as the expression of a specific type of protuberance, which is realized neither at the donor nor at the acceptor cell.

**Figure 11:**
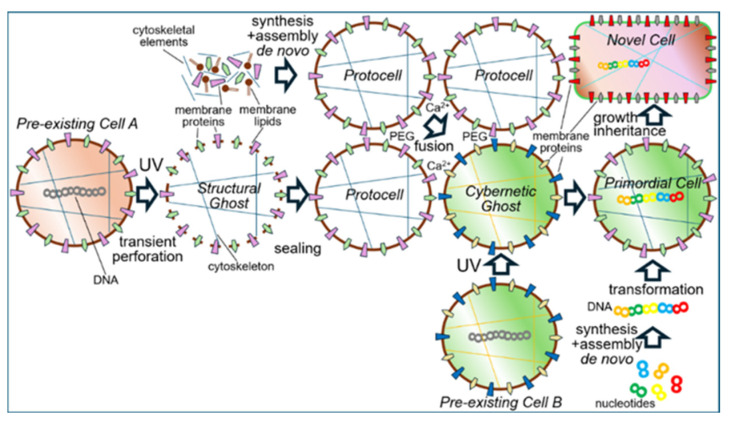
Principle of the creation of novel cells by “recombination” of DNA, macromolecular, topological, cybernetic, and cellular heredity. Pre-existing cell A, equipped with macromolecular and structural information, is depleted of DNA by UV irradiation (260 nm) and of cytosol by transient perforation [213]. This leads to “structural ghosts” still harboring remnants of their PM (e.g., membrane proteins, residual membrane lipids) as well as an intact cytoskeleton, which, upon sealing of their PM, are converted to protocells. Alternatively, protocells may be engineered in the course of de novo synthesis and assembly of their component membrane lipids, membrane proteins, and cytoskeletal elements. Pre-existing cell B, equipped with cybernetic information, is depleted of DNA (see above). The resulting “cybernetic ghosts” are then fused with protocells at very low stoichiometric ratio using various methods (e.g., Ca^2+^, polymers, PEG; for a review, see Refs. [214,215]) leading to primordial cells, which display the macromolecular, structural and cybernetic information of pre-existing cell A and B, respectively. Upon transformation with DNA, which is synthesized and assembled from its building blocks de novo, the primordial cells grow to novel cells, capable of inheritance of major features and large differences, which distinguish them from the pre-existing cell A as well as B.

The hypothetical interplay of DNA, topological and cellular heredity on the basis of the sequential integration of DNA, membrane proteins, membrane lipids, cytoskeletal and other structural elements and cybernetic fluxes of metabolites and regulatory molecules under the control of regulatory circuits and feedback loops may result in completely novel cells and is schematically presented (Figure 11).

The novel cells will continuously grow and stably inherit not only those major features and large phenotypic differences in novel combination and altered configuration, which they have acquired from the pre-existing cells. In addition, and of greater importance, in both quantitative and qualitative regard, unexpected alterations in morphology based on structural information and its topological heredity, as well as in physiology based on regulatory information and its cybernetic heredity, should emerge and merge to cellular heredity. The outcome of the fusion or hybridization is not explained by the morphology and physiology of the counterparts alone as manifested in the pre-existing cells and therefore cannot be predicted at all. A considerable increase in morphological and/or physiological diversity and complexity of those newly created cells may be achieved by the implementation of inorganic materials, resulting in so-called cyborg cells.

### 3.3. Designing Cyborg Cells

Cyborg cells are living (bacterial) cells that have been interfaced with the (i) direct deposition of magnetic and noble metal nanoparticles at, (ii) coating with polyelectrolytes and synthetic polymers of, and (iii) polymer-assisted assembly of complex nanomaterials and hard mineral shells on the cell surface [216]. Thereby, special functions are accomplished that completely differ from the specification of the original bacteria. Those functions may encompass the operation of tiny robots of cyborg bacterial cells, which have incorporated a synthetic skeleton with the aid of polymer-assisted assembly of certain nanomaterials [217]. Architectural engineering of living cyborg bacteria may also be fostered by the intracellular deposition of synthetic materials, such as the assembly of PEG-based hydrogel in their cytoplasm. This procedure apparently enables the control of their physical and biochemical characteristics using a wide variety of structurally different hydrogels. Moreover, the intracellular hydrogel-stabilized topology and architecture of cyborg bacteria can be modified upon use of the photoinitiater, PEG-diacrylate, of various sizes and its four-arm and double-strand DNA variants. This will cause modulation of their generation and metabolism as well as control of the level of their protein expression via post-transcriptional mechanisms and sequestration of the DNA-dependent RNA polymerase. Thus, modulation of the intracellular gel state, affecting the “fluidity” of both cytoplasmic and membranous components, can be used to control the architecture and function of cyborg bacteria [218].

Most recently, an experimental protocol has been reported to overcome the limitations provoked by the complexity of the cellular architecture. It is based on the construction of a novel so-called live-cell chassis combining the functionalities of natural cells and the artificially engineered simplicity of synthetic materials. It describes the introduction and assembly of an artificial network consisting of synthetic polymers in the cytoplasm of bacterial cyborg cells. It only requires the preparation of gelation reagents, hydrogel polymerization chemistry, and appropriate chassis strains, including knowledge about synthetic regulatory circuit engineering. Its application will result in important advantages with regard to resistance towards environmental stress factors, such as hydrogen peroxide and high pH or temperature, which would negatively impact the survival of natural, i.e., unmodified bacteria. Bacteria are thereby converted to precisely controlled living devices of single lifespan with programmable functionality, including but not restricted to biotechnological and biomedical engineering and the fabrication of living therapeutic agents [219].

By nature, the intracellular hydrogelation renders those cyborg bacteria as living cell-synthetic material hybrids incapable of cell division and auto-replication. However, essential cellular functions, including metabolism and protein synthesis, which enable their novel functionalization, are fully maintained. Consequently, the newly acquired features of the cyborg bacteria, as have been constructed so far, are not replicated in the donor cells and subsequently transferred and phenotypically expressed in the acceptor cells, i.e., they resist the—from the viewpoint of biotechnological application often desired—phenomenon of inheritance. However, it is conceivable that this criterion does not represent a principal limitation for the generation of cyborg bacteria. Replication and growth of (i) DNA, i.e., inheritance of the capability for protein synthesis, (ii) non-DNA matter, including bacterial prions, micelle-like LP/LPS complexes and OM/PM vesicles, i.e., macromolecular inheritance, (iii) biological membranes and intracellular compartmentalization, i.e., structural inheritance, and (iv) regulatory (feedback) circuits, turnover and metabolic fluxes, i.e., cybernetic inheritance, could be extended to artificial substances (materials) and shapes (information) rather than be restricted to their natural living counterparts.

In consequence, there is no reason to assume a priori that inheritance, as one of the commonly accepted and principal criteria for the definition of life, is incompatible with the fabrication of cyborg bacterial cells. Rather, coating of their cell surface with synthetic polymers and nanomaterials [216] or introduction into their cytoplasm of a synthetic skeleton [217] or hydrogelation of the intracellular architecture [217,218,219,220] may support the creation of novel cyborg cells lacking hereditary potential. However, and even more attractive, materials and information other than those used so far have to be selected and tested in future efforts to produce cyborg bacteria capable of cell division and inheritance.

In conclusion, the creation of artificial cells displaying true novel physiological and morphological features rather than mere subtle differences to already existing ones will necessitate both the robust integration and the fine-tuned coordination of DNA/protein, macromolecular, topological, cybernetic, and cellular heredity. The mere differential engineering of the expression of a single gene or even a complete genome will certainly not be sufficient. This major goal of synthetic biology seems to be in reach with both natural and cyborg (bacterial) cells.

So far, the following approaches have been designed to produce novel acceptor cells which did not result from the transformation of donor cells by genomes, irrespective of whether being partially or completely synthesized de novo.

(i) The binding of RNA bases and sugars to decanoic acid led to stable (i.e., non-flocculating by salt) aggregates of a prebiotic amphiphilic membrane-like structure (in additive fashion) which may support the emergence of viable protocells by mutually reinforcing mechanisms through their stabilization towards salt [221];
(ii) A protocell-like supramolecular assembly, called “Jeewanu”, was prepared by sunlight exposure of an aqueous mixture of inorganic and organic substances, which, upon structural analysis (based on acidic and basic dyes), revealed a regular organization with metabolic characteristics and the ability to convert solar energy into useful forms. These results strongly argued for the existence of similar energy-transducing systems in a prebiotic atmosphere [222];
(iii) Molecular systems were developed by cycles of hydration and dehydration, undergoing chemical evolution in dehydrated films on mineral surfaces, followed by encapsulation and combinatorial selection in a hydrated bulk phase. This led to the formation of a dehydrated phase consisting of concentrated eutectic mixtures or multilamellar liquid crystalline matrices and concomitantly of vesicles. Each of them represented a protocell during an “experiment” in a natural version of combinatorial chemistry, which, upon continued cycling over time, may result in molecular systems having fundamental properties of life [223];
(iv) Efficient and stable systems have been introduced undergoing structural reproduction, self-optimization and molecular evolution whereby (iv-a) two cyclic processes interact, one for vesicles as structural environment, the other supplying peptides from various amino acids as versatile building blocks; (iv-b) combination of both cycles support their own existence to undergo chemical and structural evolution; (iv-c) unpredicted functional properties may develop; (iv-d) mutual stabilization of the peptides by the vesicles and of the vesicles by the peptides with constant production of both occurs; (iv-e) combination of both cycles serve as a model for the formation of self-evolving structures, leading to the first living cell; (iv-f) vesicle-induced accumulation of membrane-interacting peptides happens, which results in a reduction in vesicle size, increase in membrane permeability and improvement of vesicle thermal stability [224];
(v) It has been confirmed that interactions between RNA/DNA and proteins act as cornerstone of biological processes [225], such as gene regulation, among them the binding of short RNAs (≥3 n) in sequence-dependent manner to peptide amyloids with (v-a) 3′-5′ linked RNA backbone supporting these (electrostatic) interactions; (v-b) phosphodiester backbone and nucleobases (negatively charged) and amyloids (positively charged) contributing to affinity (differences between RNA and DNA); (v-c) support of self-replication of the amyloids; (v-d) mutual increase in stability of both RNA and amyloids; (v-e) local increase in the concentration of RNA/DNA in diluted unordered systems; and (v-f) selective (evolutionary?) advantage of cooperation vs. competition for interacting molecules [225];
(vi) Artificial cells, based on non-replicating materials, with reduced biochemical complexity but upregulated, more defined, and controllable functions, were constructed. These efforts resulted in the first report on the creation of hybrid material-entities termed “Cyborg” cells [220]. The assembly of a synthetic polymer network inside each bacterium rendered them incapable of division, too, however, with preservation of essential functions, among them cellular metabolism, motility, protein synthesis, and compatibility with genetic circuits. Furthermore, the acquisition of new abilities was aimed at the resistance towards cellular stressors and necrosis/apoptosis, as well as at invasion into cancer cells, with a combination of intracellular man-made polymers and their interaction with the protein network of living cells [220].

Those findings may be interpreted in alternative fashion as the construction of true artificial (pro- or eukaryotic) cells by the combination of (partially or completely) synthetic genomes and protocells, lacking an own genome, as (i) construction of a protocell de novo, i.e., through self-assembly rather than self-organization, (ii) emergence of protocells which eventually do not rely on typical biological membranes (and consequently on any enclosed aqueous compartment) and do depend on silicon rather than carbon chemistry, (iii) “fitting” between the novel genome and the (modified or novel) acceptor protocell, and finally (iv) emergence of unexpected novel features characteristic of true artificial cells.

The greatest challenge in the creation of both natural and artificial life is commonly regarded as the development of biological membranes and intracellular compartmentalization during evolution and the synthetic biology approach, respectively. Thus, the initial stage in the origin of cellular life on earth is thought to rely on the self-assembly of free fatty acids in a stable configuration, i.e., vesicles, fostered by the binding of building blocks of RNA, such as nucleobases and sugars, and protein, such as amino acids [226]. Those may lead—via the selection and concentration of prebiotic cellular components—to stabilization and expansion of membrane precursors. Membrane binding of prebiotic components may induce–via conformational constraints and altered chemical environments—the synthesis of nucleosides, oligonucleotides, and peptides. It is hypothesized that the oligomers formed, even if of short and random nature, could have improved the stability and growth of vesicular and membranous structures to a greater degree compared to the individual building blocks. Those vesicles might compete with each other, resulting in the emergence of (extended) polymers during the early evolution of life, which are engaged in functions of higher complexity [226].

For the area of synthetic biology, the bottom-up construction of a minimal cell is considered to represent a feasible and realistic goal, despite the necessity to tackle a multitude of challenges. One of the most important is the integration of individual membrane-based sub- and micro-compartments. It relies on complex and finely tuned interactions between diverse self-organization and self-assembly processes, which are required for the generation of a growing and self-reproducing/regenerating cell, capable of inheritance. It must encompass mechanisms of protein synthesis and microfabrication of all other essential building blocks and biobricks [227]. The recent demonstration of partial molecular self-regeneration in a synthetic cell may be regarded as a key step for the development of a minimal cell. By the integration of a minimal coupled transcription-translation system into microfluidic reactors, the regeneration of essential protein components initiated by DNA templates and sustained by ongoing synthesis was achieved for considerable periods of time (>24 h). Analysis of quantitative genotype–phenotype relationships in combination with computer modeling revealed that the minimization of resource allocation and optimization of resource competition is most critical for robust stabilization of the function of that system [228]. This was reflected in the simultaneous regeneration of various polypeptides, which was monitored by determination of the DNA ratios required for continuous self-regeneration. This study may provide the theoretical and experimental basis for the future construction of a synthetic self-replicating and growing cell [228].

Together, these experimental approaches go far beyond the mere exchange of genomes, synthesized partly or completely de novo, between prokaryotes (for a review, see Refs. [229,230,231,232]) as well as eukaryotic unicellular organisms. Eventually, with appropriate modification, it could be extended to metazoans (for a review, see Refs. [233,234]). Importantly, the situation cannot be compared with frozen unicellular organisms. Those have been operational before freezing, with respect to protein synthesis, biogenesis of membranes, compartments, other structures, metabolism, and other vital functions. And the trigger of the “heart” of those frozen cells to “beat” for the first time upon thawing relies solely on their “history” or “tradition” as former fully functional and vital cells, according to Virchow’s dogma “omnis cellula e cellula”.

## 4. Conclusions–Creation of Novel Microorganisms by “Facilitated Variation”

### 4.1. Early and Present Conceptions About the Relationship of DNA, Non-DNA Matter, and Cells

**Figure 12:**
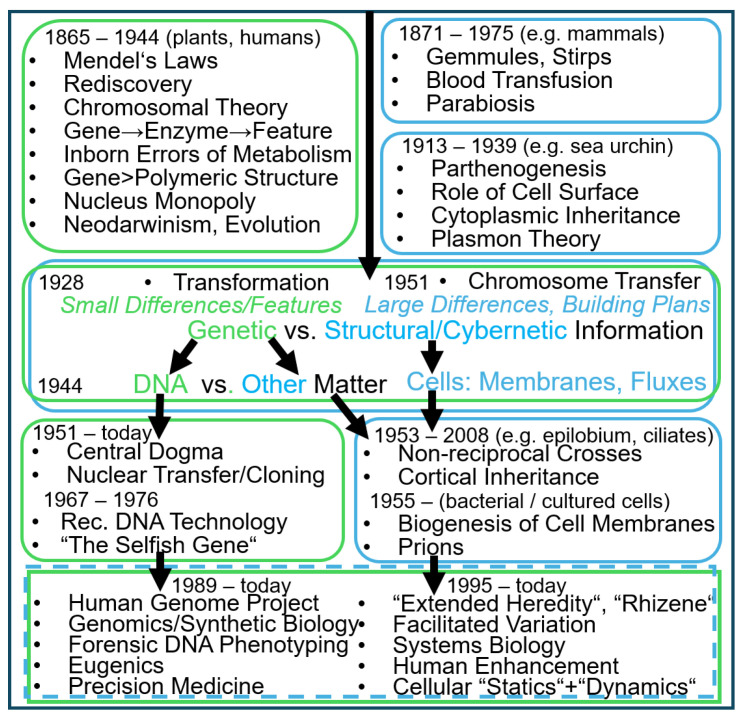
Historical narratives for the explanation of the emergence of small differences/minor features vs. large differences/major features (e.g., building plans) as the transfer of information for the synthesis of proteins/macromolecules (green boxes) and structural/cybernetic information for the assembly of membranes/organelles and regulation of metabolism, turnover and fluxes (blue boxes), respectively. Prior to the Griffith transformation experiment (design 1.0 and 1.1) [17] and chromosome transfer [39], the dichotomy or agential cut between the nucleus monopoly or neodarwinian synthetic evolutionary theory and the “plasmon” theory or cytoplasmic inheritance was initiated by the (re-)discovery of the Mendelian laws, on one side, and the postulation of the operation of “gemmules” [235,236,237] and “stirps” [238,239] on the basis of blood transfusion [240,241,242,243,244] and parabiosis [245,246] experiments, on the other side (for a review, see Ref. [247]. Thereafter, the central dogma of molecular biology till the conception of “The Selfish Gene” [199] as well as the description of cytoplasmic to up to cortical inheritance (for a review, see Refs. [248,249,250]) to up to the study of the biogenesis of cellular membrane systems, prions and intrinsically disordered proteins [251,252,253] contributed to further stabilization of the apparent incompatibility between nuclear and non-nuclear inheritance. This dichotomy or agential cut remained manifest in the typical DNA-centric views, such as the human genome project, eugenics, and precision medicine vs. the holistic or pluralistic conceptions of “extended heredity”, “rhizene agency”, and “facilitated variation” (for details, see below) till the midst of the 1990th. From then on, some “permeabilities” between them or “openings” for certain aspects of their counterparts (as visualized by the hatched blue box) resulted in the acknowledgement of interactions or “intra-actions” or “cutting things together-apart” [254] of genetic/macromolecular and structural/cybernetic information, substance/matter and form/information, DNA and non-DNA matter, DNA and topological/cellular heredity rather than emphasizing their dualistic or binary “nature”. Representative narratives of major impact are given, only (albeit their presentation here is far from being complete).

The following synopsis represents a compilation of historical key findings for the emergence of the two-fold dichotomies between genetic/macromolecular and structural/regulatory information, as well as between DNA and non-DNA matter of inheritance. They critically affected conceptions of natural evolution as well as synthetic biology approaches for the creation of novel (micro)organisms (Figure 12).

The following key narratives with relevance for the emergence of those dichotomies or “agential cuts” are explained in greater detail (see Figure 12).

(i) Theory of “Gemmules” and “Stirps”. Charles Darwin developed his “pangenesis” hypothesis more than 150 years ago to support his theory of inheritance, species diversity, individual variation, and the development of organisms [235]. For this purpose, he introduced molecules of heritability and heredity and called them “gemmules” [236,237]. “Pangenesis” was based on the idea that all parts of the body produce units, which were able to replicate by self-division, and after being transported to the sexual organs, were instrumental to heredity and development. According to his conception, those “gemmules”, which were at the time not clearly distinguishable from cells, are produced and released by all parts, organs, and cells of the organism and then accumulate in or near its germ cells due to mutual affinity. However, they do not represent a simple image or representation of the parts, organs, and cells that have produced them. After transfer from the gametes to the zygote, the parts, organs, and cells that were originally responsible for their production and release are formed from the “gemmules” during development of the zygote to the adult offspring. As a consequence of some ill-defined forces, which may have been characterized during later periods as self-organization and/or self-assembly, the “gemmules” apparently succeed in the reconstitution of all functional parts, organs, and cells in the emerging organism. Thus, “pangenesis” is compatible with both preformistic and epigenetic conceptions of development.

The assumption, that according to Darwin [235] the “gemmules” from the modified units will be themselves modified, and, when sufficiently multiplied, will supplant the old “gemmules” and be developed into new structures, made it possible to explain the direct action of altered environmental (f)actors and of the increased use or disuse of bodily parts. With this hypothesis, Darwin finally established the theoretical foundation for a mechanistic explanation of “soft” or “Lamarckian” inheritance of acquired traits. Despite the apparently discrete nature of the “gemmules”, the fact that an unknown large number of them of all possible sizes, produced during the complete lifespan, accounted for an unknown number of phenomena, blurred any kind of discrete effects, and favored the occurrence of quantitative, i.e., infinitesimally small variations. “Pangenesis” thus supported essential conceptions and claims of Darwin, in particular inheritance by “blending”, inheritance of acquired characteristics, and the hereditary effects of use and disuse.

Francis Galton intended to demonstrate the validity of “pangenesis” based on blood transfusion experiments (between rabbits of different coat colors). Unexpectedly, he finally failed completely [238,239]. This was possibly due to the experimental design used, as has been suggested more than 80 years after its performance on the basis of a valid calculation of the putative dilution by the blood of the acceptor animal. Other, but not all, researchers reported on seemingly successful efforts of reproduction of “pangenesis” [245,246]. Nevertheless, because of Galton’s negative results, “pangenesis” was immediately assessed as being completely obsolete. And this view has been kept in the (scientific) communities of geneticists and (developmental) cell biologists as well as animal and plant breeders for the next period of 70 to 80 years.

Interestingly, Galton tried to rescue parts of the “gemmules” conception and therefore suggested that individual “gemmules” constitute so-called “stirps”, which reproduce truly themselves [240]. However, which type and how many of the “gemmules”, he conceived to be contained in the “stirps”, develop [241] “*depends on their position in the overall structure*” of the “stirps”. This also held true under the assumption that they are engaged and survive in the resulting interplay of forces against the other “gemmules” of the “stirps” (i.e., survival of the fittest “gemmule”). On that basis, Galton explained, e.g., the fact that siblings can considerably differ from one another, as well as that certain features can skip a generation and become manifest in the parental and grandchildren rather than children generation, despite the educated guess that their respective “stirps” should be quite similar in composition and structure [242,243,244].

Taken together, “gemmules” and “stirps” as particles, being released from all organs and tissues and thereafter–in the course of their replication–re-assembled to the same organs and tissues from which they had been liberated, could be regarded as (sub)cellular entities with inherent structural and cybernetic information, supporting topological and cellular heredity.

(ii) Cytoplasmic Inheritance. In the first third of the last century, most biologists examined their test subjects using Mendelian genetics, which was aimed exclusively at recording nuclear genes. The cytoplasm was not considered to play a primary role in heredity. It was the time of the nuclear monopoly of heredity. However, individual botanists working cytogenetically gradually raised serious doubts about this one-sided view and extended their investigations to the cytoplasm. Then, in the 1930s, the German botanist Peter Michaelis used breeding studies to investigate heredity on the basis of a method exclusively adapted to the cytoplasm, the so-called reciprocal crossing with up to 13 backcrosses (*Epilobium hirsutum* x *Epilobium luteum*) [248,249,250,255]. In doing so, he succeeded in transferring the largely homozygous nucleus or genome of one *Epilobium* species into the alien cytoplasm of a second species, thus providing decisive proof that “*the plasma had retained its independent genetic properties over eight generations*”. Michaelis was thus able to prove that plant breeds differ not only in their genome, but also in the genetically independent cytoplasm [256].

Heinz Brücher also strengthened the general opinion that, in addition to the cell nucleus, the cytoplasm plays a critical role as a carrier of heredity [257]: “*In these cases, a genetically independent plasmon is added to the genetic material of the genome in the cell nucleus*”. Cytoplasmic inheritance as a form distinct from nuclear inheritance was thought to rely on hereditary constituents localized in the “plasmon” [258], “*which is expressed in the control or co-control of certain characteristics by plasmic hereditary factors*”. Accordingly, “plasmon” was used as the collective term for all extrachromosomal inherited cellular elements of the cell [259]. It was divided into two components: (i) the “cytoplasmon” with the cytoplasmic carriers and (ii) the “plastome” with the plastid elements. Subsequently, this resulted in the emergence of the “plasmon” theory.

(iii) “Plasmon” Theory. Fritz von Wettstein understood the “plasmon” to operate as a genetically active “*homogeneous plasma mass*” without the comparable individual elements of nuclear genes or plastids, which ensure the correct arrangement of the various cellular components in three-dimensional space and time [260,261]. Heinz Brücher also strengthened the general opinion that, in addition to the cell nucleus, the cytoplasm plays a critical role as a carrier of hereditary constituents localized in the “plasmon” [258,259]. Fritz von Wettstein understood the “plasmon” as ensuring the correct arrangement of the various cellular components in space and time [262].

Orthodox “plasmon” theoretics, such as Werner Zündorf, considered it to be a stable, uniform cytoplasmic structure that is involved in the constitution of all features, so that there are “plasmon” equivalents for all genes in all organisms [263,264]. Radical critics did not believe in the “plasmon” at all and supported the nuclear monopoly. Within this dichotomy, so-called revisionistic “plasmon” theoretics accepted the existence of the “plasmon” but considered it less important for the explanation of heredity processes. Therefore, they invoked it only sparingly, often doubted its stability, and/or considered its nature to be probably particulate.

Taken together, the previous understandings of the term “plasmon” should be broadened to combine the principles of topological/cybernetic heredity and macromolecular heredity as based on non-DNA matter, such as prions and intrinsically disordered proteins [251,252,253], micelle-like GPI-AP and LP/LPS complexes, and PM/OM and extracellular vesicles, to critically contribute to cellular heredity.

(iv) Structural Inheritance. Structural inheritance refers to the transmission of alternative three-dimensional structures in a living organism through self-perpetuating spatial templating: a variant structure in a mother cell guides the formation of a similar structure in a daughter cell, leading to the transmission of the architectural variant. An early example was the inheritance of variations in the cortical structures of ciliates [265]. This contrasts with the sole transfer of DNA as a form of digital information, which is responsible for the propagation of the majority of polymorphisms and mutations. Structural inheritance has been described for the orientation of cilia in protists, such as *Tetrahymena* and *Paramecium*, as well as the “handedness” of the spiral of the cell in *Tetrahymena*, and shells of snails [266]. Moreover, certain organelles support structural inheritance, such as the centriole. And most importantly, the cell itself, as it is surrounded by PM, can be regarded as a complex arrangement of (PM) molecules, which is transmitted by structural inheritance.

More than 60 years ago, the American biologist Tracy M. Sonneborn introduced the ciliate, *Paramecium caudatum*, as a genetic model organism [267]. Based on a variety of different spontaneous or experimentally induced “mutations” in the cortical organization of *Paramecium*, he succeeded in the demonstration of their hereditary persistence in the course of cellular continuity. This was not associated with any change in the DNA-encoded information. Sonneborn interpreted this observation of “cortical inheritance” in terms of the concept of “cytotaxis”, “structural templating”, or “structural inheritance”, considering the critical role of pre-existing structures and arrangements on the assembly of novel structures and arrangements [268,269,270]. This was found approximately at those times when the discussion about the scrapie agent was started as representing a non-DNA-based entity which follows a novel non-DNA-based mode of inheritance [271].

In conclusion, (i) there is no one-to-one correspondence between genotype and cellular architecture, and (ii) the directing role of the pre-existing structures on the assembly and arrangement of new structures argues for the transfer of a novel type of information that is based on non-DNA matter. At those times, Sonneborn and colleagues did not introduce the term “protein-based inheritance”, but they did insist that DNA in the basal bodies or other cortical components was not responsible for those observations. They also were not caused by the overall characteristics of the basal bodies or associated components, but rather by changes in the three-dimensional arrangement of those constituting elements, which themselves remain unchanged at the molecular level [265]. (iii) During his investigation of *Paramecium* in the late 1930s, Sonneborn found that the structure of the cortex did not depend on genes or the cytoplasm but was severely affected by the cortical surface structure of the ciliates [265,272]. The structure of pre-existing cell surfaces represented a template which was transmitted from one generation to the next for many generations. Sonneborn concluded that the specific configuration of surface structures within macromolecular complexes is inherited.

Since then, a large body of evidence has accumulated for the only major surviving concrete examples of non-genetic cytoplasmic inheritance, i.e., for *Paramecium* and *Tetrahymena*. In fact, geneticists have long been aware of the operation of mechanisms responsible for the inheritance of cortical patterns but have downranked their meaning and frequency as an exception to the general rule of genetic inheritance. In contrast, embryologists, who were predominantly interested in the development of organisms rather than in the transmission of genes between them, did not agree upon the hegemony of genetics over cytoplasmic non-genetic inheritance. Consequently, some of them [272,273] postulated that both DNA and cortical inheritance represent components of “*directed assembly wherein the timing and placement of new structures are organized according to a template of pre-existing structures*”.

Based on the identification of “cortical mutants” displaying inverted patterns of their cortex, which become stably inherited for hundreds of generations, it has been recognized that the genes encoding the cortical proteins are the same between the generations, but that the latter become organized in different configurations. Even today, the exact molecular basis of cortical inheritance is not entirely clear, but it seems to be based on self-templating of complex molecular structures, similar to the transmission of the characteristics of macronuclei to new macronuclei [273,274].

Thus, the validity of the conception of cortical inheritance cannot be questioned, since developmental biologists, among them Sonneborn, Beisson, and their colleagues, did a great job in convincing other researchers of its operation, based on successful experiments in protozoa. This led to the urgent question of whether protozoa represent exceptions to the general rule or examples of the general rule. Behave protozoa “normally” or “exceptionally”? According to Grimes and Aufderheide, all biological membranes are of composite, mosaic, and complex nature, based on the insertion of novel constituent components into the pre-existing membrane structures. Thus, it is tempting to speculate about the extrapolation of the inheritance principle realized in ciliates to eukaryotic cells in general. However, the fluid mosaic model as proposed for the membranes of metazoans must not be mixed up with the highly structured and complex cortex at the cytoplasmic periphery underlying the PM of ciliates. Consequently, Scott F. Gilbert summarized the state of the exception-rule debate at those times and concluded that [275] “*until stably inherited membrane structures are discovered in such metazoan cells (and not merely transient organizations…..), cortical inheritance is likely to remain at the periphery of discussions on inheritance and development*”.

According to a more fundamental view, the phenomenon of cortical inheritance represents a reminder to us that [276] “*the fundamental reproductive unit of life is not a nucleic acid molecule, but the remarkable versatile, intact, living cell*”. Further analysis of the phenomenon of structural inheritance has meanwhile been incorporated into the theory of the extended heredity and evolutionary synthesis [277,278,279,280,281,282,283,284,285,286,287,288].

(v) “Rhizene Agency”. In their book “A Thousand Plateaus”, published in 1987, the French philosopher Gilles Deleuze and psychoanalyst Felix Guattari proposed the biological phenomenon of the rhizome as a metaphor for a network that allows connections from any point to any other point at any time without any hierarchy [289]. The possibilities of connections in all directions and the consequent dissolution of the hierarchical order offer an approach of thinking about relationships everywhere and always (e.g., between genes, or DNA and non-DNA matter or DNA and structural/cybernetic information or DNA and topological/cellular heredity or scientific communities and society) that reflect the apparent structural and regulatory disorder underlying successful long-standing rhizomic plant communities.

If a single rhizome is planted with a lot of space around it, it will soon naturally form its overlapping network of connected structures. It is the way it grows. Using language as one of their first examples in their poststructuralist rhizomic analysis, Deleuze and Guattari move fluidly between biological facts about rhizomic plants and metaphorical extensions, noting that [290] “*there are no points or positions in a rhizome like those found in a structure, tree, or root. There are only lines*”. The knowledge tree is a model of order that is intended to make hierarchies of knowledge and sciences describable. Tree models are both hierarchical and dichotomously oriented, which means that each element is on one (and only one) level of order, is subordinate to a higher level, and can be superior to one or more elements. There are no cross-connections that skip hierarchy levels or connect elements that are superior to two different higher-level elements. A rhizome is therefore a “multi-rooted”, intertwined system that cannot be depicted in simplistic dichotomies. Rhizome also means liberation from defined power structures. Many perspectives and many approaches can be freely chained. Thus, for Deleuze and Guattari rhizome serves as a metaphor for a postmodern model of knowledge organization and world description that replaces older hierarchical structures represented by the tree metaphor [290].

The rhizome metaphor was received in the philosophy of science, the philosophy of science, the philosophy of media and cultural studies with great interest. It rejects hierarchies and thus the conception of the traditional form of the tree of knowledge. Many modern media theorists, therefore, consider the metaphor of the rhizome to be suitable for describing the structures of hypertexts, social networks, or computer networks such as the Internet, but also conceptions of heredity.

Here, I argue for the broadening of the use of the term rhizome in order to combine in a non-hierarchical fashion the conceptions of DNA, structural and regulatory information, macromolecular, topological, cybernetic, and cellular heredity, DNA and non-DNA matter, prions and intrinsically disordered proteins, micelle-like GPI-AP and LP/LPS complexes, extracellular and PM/OM vesicles. Accordingly, the term “rhizene”, a neologism between rhizome and gene, and the corresponding conception of “rhizene agency” is considered as the concerted and continuous inter-/intraaction between DNA, genes, nuclei, “gemmules”, “stirps”, cytoplasm, “plastom”, and other cellular structures/compartments to enable replication and transfer of major features and large differences.

(vi) Interpersonal and Intercellular “Communicology”. It could be revealing for an understanding of one of the major (but certainly not the only) (f)actor which exerted the exclusion of structural and cybernetic matter and information from macromolecular, topological and cellular heredity and concomitantly resulted in the DNA-centric bias of inheritance in the course of the past 80 years to draw some surprising analogies between our reception of the biological transformation and inheritance processes and the development of media and defined stages of evolving mediumship during the history of mankind. The latter has been developed by the Czech-Brazilian philosopher of media and scientist of communication Vilem Flusser from the end of World War II till his death in 1991. The (f)actor of human communication and its adherence to linear thinking and alphanumeric writing has not been addressed in previous publications by me [16,24,291], and according to my knowledge in recent STS as well.

The following principles characterize Flusser’s conception of communication: (i) According to Flusser, human communication is “unnatural”. Their artificiality is particularly evident in their symbolic mediation (for a review, see Refs. [292,293]). Flusser does not understand the accumulation of information as a process that can be derived from laws, but traces it back to the human will, i.e., ultimately to its freedom. (ii) The incarnation must be understood—in the sense of existentialism—as alienation: man is expelled from the world (or springs from it), i.e., he is no longer absorbed in it like the animal but confronts it as alien. In being thrown, there is thus a feeling of powerlessness, of forlornness, of loneliness. (iii) Symbols are intended to bridge the gap that reflection opens up between the world and man by signifying the world. They intend to represent the world, structure it, and provide orientation. According to Flusser, however, symbols are dialectical in nature, i.e., a symbol not only has the potential to represent the world, but it also has the tendency to cover the world. For Flusser, this finding addresses the possibility that a code obscures more than it signifies. (iv) After all, according to Flusser, communication is one of the decisive factors for our position in and to the world. Symbols are not to be understood as mirrors of reality; they do not merely depict the world, as is claimed in typical philosophical theories of representation (see the early Ludwig Wittgenstein), but always undertake a construction in their abstract depiction and accentuating description. According to Flusser, this construction is not independent of the type of symbols and thus language, but rather that each code has a typical imprint and thus a way of life (for a review, see Refs. [292,293]).

Flusser’s concept of media aims at a typology of forms of objectification and representation that replace each other historically [294]. The five media epochs correspond to four stages of cultural development, which are preceded by an early and “medialess” precursor to the merely animalistic-reactive being-in-the-world. Flusser speaks of a preluding “stage of concrete experience”.

This first epoch can be assigned to a natural person who lives in a four-dimensional environment of immediate and “concrete experience” in a phenomenological sense as magical consciousness in space-time. This may also have encompassed the holistic experience about the phenomenon of inheritance, including major features and large differences between man, animals and plants, in general, and may have been expressed in the “amazement” about identity (e.g., twins) and “admiration” of similarity (e.g., successive generations, breeding successes), particular.

Out of it develops the first stage of culture and mediality–the way of life of the Upper Paleolithic people, who appropriate the world through tools and weapons, dwelling and clothing, and reshape it (and themselves). The characteristic of this level is the first “interest in objects” that can be grasped, handled, and shaped. This stage, therefore, refers to the interest of the human being in “objects”, i.e., in that of a three-dimensional environment. It can be thought of as a representational (=“three-dimensional”) reference to reality. Although the objects function as media, they are not yet understood as media. According to Flusser [294], it is only through such an understanding that prehistory passes into history. Only now has the so-called prehistory come to an end. This receding of the subject in front of the object, this observation from an elevated position, necessitates the formation of cracks as a prerequisite for “grasping”, i.e., also for the analysis of cellular heredity with regard to the distinction between cybernetic, structural, and macromolecular heredity.

The following second stage is considered the “stage of traditional images”–the Upper Paleolithic cave painting about 30,000 years ago is considered a caesura. As the first medium that is understood as such, the images place themselves between people and the world and establish an interplay of vividness (=proximity) and artificiality (=distance) in human thinking on the one side and world behavior on the other. The reality reference of this level is “two-dimensional”. This also includes the present models and analogies for making macromolecular heredity and the materials and information transmitted thereby, e.g., DNA, proteins, EVs, micelle-like complexes, prions, “tangible” or “imaginable” long before any scientific argumentation.

Flusser [294] calls the third stage—caesura is the (roughly simultaneous) invention of writing and numbers about 5000 years ago—the “alphanumeric” or “linear text” stage. What letters and numbers have in common is the need to represent them sequentially or linearly. According to the view presented here, this holds true for the present description of DNA or proteins as nucleotide or amino acid sequences, which goes hand in hand with an abstract and “one-dimensional” way of thinking and conception of macromolecular heredity. Communication to the world no longer takes place via concrete, descriptive images, but through the translation of the pictorial into a conceptual-theoretical reference to the world. This also refers to all the knowledge about heredity which has accumulated during the literal period till and including the deciphering of the genetic code, among them the formulation of Mendel’s law of inheritance, the pedigree analysis of human diseases by Archibald Garrod, the synthetic theory of evolution and genetics postulated by Hugo de Vries, Ernst Mayr and Theodosius Dobzhansky, the transformation experiment performed by Griffith with lethality of mice as visible, countable and writable phenotype (see Figure 1 and Figure 2), the identification of DNA as matter of inheritance by Avery and coworkers with the staining and corresponding classification of (R) and (S) bacteria as visible, countable and writable phenotypes (see Table 1) and the demonstration of DNA transfer between bacteria (chromosome transfer) by Lederberg and coworkers with selection for prototrophic vs. auxotrophic mutant colonies as visible, countable and writable countable phenotype [16]. All the relevant data in the corresponding reports and publications were presented in letters and numbers, i.e., in alphanumerical codes, in some instances illustrated by traditional images and cartoons, i.e., two-dimensional codes.

The fourth and last stage—telematics—is the “level of techno-images”, to which Flusser [294] ascribes a “zero-dimensional” reference to reality. These new telematic images no longer represent “images of reality”—like the “traditional images” of cultural level two—but are constructed and “computated” (=artificially composed of abstract points or pixels). “Zero-dimensionality” means that there is no longer any reference to an objective human-independent reality and that it is replaced by the freely acting subject—inventing artificial worlds and playing with the artificial worlds. This subject is not autonomous in Kant’s sense, but socially and technically dependent and constructed in many respects. The dependence is expressed in its digital networking and complex relationships mediated by a variety of apparatuses and machines for the generation of the corresponding new non-alphanumerical codes. With regard to research on biological inheritance, the introduction of the method of radiolabeling of biological materials, such as DNA and proteins, and subsequent identification and quantification of the radiolabeled materials by appropriate apparatuses, such as the Geiger—Müller counter, radioactivity liquid scintillation counters and autoradiography systems, with connected plotters and imagers, led to the use of techno-images as manifested initially in the experimental demonstration of the infection of bacteria by DNA rather than protein contained in bacteriophages by Alfred Hershey and Martha Chase (Geiger—Müller counter; for a review, see Ref. [16]) and subsequently in the methods of deciphering of the genetic code by J. Heinrich Matthaei and Marshall W. Nirenberg (liquid scintillation counter) and of sequencing of DNA by Allan Maxam and Walter Gilbert as well as Frederick Sanger, S. Nicklen and A.R. Coulson (autoradiograms).

In conclusion, codes of the first dimension seem to be required and sufficient for the communication of the phenomenon of biological transformation without endangering or even making impossible its comprehensibility, meaning-giving function, and ability to assign meaning for the human mind. However, or precisely because of this, the code of the first dimension enables or at least facilitates communication exclusively in “black and white”, without (soft) transitions and thus favors (binary) thinking in opposites and dualisms. In fact, the phenomenon of bacterial transformation seems to correspond to a linear process: Donor bacteria transfer replicated materials and/or information to acceptor bacteria eliciting accompanying changes in the phenotype of the latter, which brought to the fore questions about the nature and materiality of the matter of inheritance in the sense of either DNA or protein. There is no need for communication via three or two dimensions since this conception excludes topological heredity with its three dimensions (i.e., arrangement of subcellular structures in space) as well as cybernetic heredity with its three + x dimensions (i.e., fluxes and turnover of cellular components, such as metabolites and currents, in space and time) and only considers macromolecular heredity with its single dimension (i.e., intercellular transfer of macromolecules, such as protein or DNA, in time). In the course of the transition to zero dimensions, apparatuses generate techno-images, such as scientific curves by plotters and autoradiograms by X-ray films, which, however, are often converted into the alphanumerical linear code, such as the genetic code or a publication thereof. It is actually conceivable that the loss of an apparent meaningfulness, the increase in complexity and the lack of transparency cause a “refolding” or “bending” of the techno-images into traditional images in the form of models (e.g., cartoons, schematic drawings) or into the alphanumeric code, which may also encompass appropriate analogies (e.g., metaphors) and images in text/text with images.

Thus, in Flusser’s work, codes and media function as instances of translation and re-translation. In “Für eine Philosophie der Fotografie”, Flusser [295] develops his idea of media history in the sense of a series of transfer processes between the two basic codes, the images and the texts. Images transcode the reality situation. Texts transfer the circular magical time of the images into the linear time of the story. The camera translates history into programs and techno-images. For Flusser, traditional images are significant surfaces that have been created by reducing the original four-dimensional space-time to the two-dimensionality of the surface. Images were invented to make ‘the world out there’ imaginable and thus understandable. Texts, on the other hand, are one-dimensional codes that were born by threading letters onto lines.

Flusser’s history of media evolution is conceived as a series of disappointments that lead to an increasing loss of confidence in the code’s ability to mediate between humans and the world. At the beginning, each new code promises to free man from the dominance of the former and to reopen the media-obstructed path to the world, but in the course of time, it becomes comprehensively determining. But with the entry into the last stage–that of techno-images–this dynamics seems to come to a standstill. The path from the original concreteness of four-dimensional space-time into increasing stages of abstraction–Flusser calls this medial escalation, which is at the same time a subtraction, the ‘abstraction game’ –seems to turn into a new concreteness of the second degree, in which duality, binary thinking and the initial rift caused by a retreat from the world are abolished by the fact that man lives in a completely artificial self-designed world and also incorporates himself into a fully aware for the first time. There is no ‘world out there’, we only encounter our own models projected into the world and rediscovered there, the codes that we have created ourselves in collective dialogs in order to find our way in the environment.

In this step model, the sign of the higher level refers to the experience of the next lower level. An image represents the three-dimensional objects represented; a text means an image, which in turn means the objects. By reducing the image content to one dimension, the text can be analyzed and copied. In the same way that the prehistoric phase of images was overcome by the historical phase of texts, post-history overcomes history through the invention of techno-images that are supposed to make texts imaginable again. By making this possible, however, post-history bends the progressive linear development of the translation of images into texts in a circular or spiraled, but in any case non-linear, manner back to its origin and at the same time beyond it. Flusser describes this process as a “*retranslation of concepts into ideas*” [295], i.e., as translation of texts containing images into techno-images containing texts. Techno-images differ from traditional images in that they are the result of different translation processes. Traditional images have real situations as their starting point, techno-images, on the other hand, are based on texts that were written to break up images through translation, i.e., images that contain texts that in turn have images in their “stomachs”. The present situation is thus not so much the result of a linear development from the idea to the concept as the consequence of a kind of spiral movement from the image through the concept back to the image.

At the level of calculation and computation into which we are currently emerging, the level of technically produced images and zero-dimensionality, a new form of concretization has become possible. The empty, abstract punctual universe, from which all dimensions have been successively subtracted, makes it possible to reach “*from the abyss of intervals to the surface*” and “*from the most abstract to the seemingly concrete*” [296] through concretizing calculation and computation. The imaginary surfaces of the techno-images stand for an apparent reality because one would need far too many points to create a concrete world. The area, which can never be reached in this way, is therefore described by Flusser as apparent. Above all, it is about a new, changed understanding of concreteness as an artificial appearance. It is not the given reality that is concrete, but the meaning projected onto the screen. You cannot live in such an empty and abstract space. In order to be able to live, one must try to concretize the universe and consciousness. One must try to gather the point elements in order to make them concrete (comprehensible, imaginable, treatable) again. In other words, you have to give the universe a meaning: the surfaces of the techno-images arise from the assembling and balancing of countless points, from an increasing condensation. “*The images stand at the outermost limit of abstraction reached so far, in the zero-dimensional universe, and they offer us the opportunity to experience the world and our lives in it concretely again*” [296].

Flusser assumes (for a review, see Refs. [292,293]) that every society is shaped by the interplay of two forms of communication, (i) dialogs that generate information, and (ii) discourses through which information is passed on. By analogy, it is argued here that the biogenesis of (sub)cellular structures is controlled by the interplay of self-organization and self-assembly, respectively.

Principally, three forms of society or cell and their corresponding cultural inheritance (tradition) and biological inheritance (stable transmission of phenotypes), respectively, between many generations can be derived from this assumption: (i) The previous “ideal society” or “ideal cell” in which dialogs and discourses or self-organization and self-assembly are in balance. (ii) The “authoritarian society” or “autonomous cell”, in which discourses or self-assembly dominate. The lack of dialogs or self-organization results in a lack of information. Discourses or self-assembly are no longer fed with information by dialogs or self-organization. (iii) The future and “revolutionary society” or new “synthetic cell”, in which dialogs or self-organization predominate, which constantly generate information. Due to the resulting flood of information, the old discourses or the existing processes of self-assembly break down. Accordingly, there are no absolute laws or rules given by mankind or nature, respectively, in the “zero-dimensional” society or synthetic cell. Due to their networked structure, societal as well as biological systems are completely opaque and direct themselves structurally and cybernetically. Thus, Flusser’s “zero-dimensional” society, and as one is tempted to add the synthetic cell, can also be described as the “cosmic brain”.

An additional essential element of Flusser’s theory of communication is therefore the fundamental dialogical and intersubjective nature of all communication. We communicate in the face of death and annihilation. Man is a zoon politikon, a political animal, as Aristotle noted, but “*not because he is a sociable animal, but because he is a solitary animal incapable of living in solitude*” [294]. Because of this unavoidable existential dimension of all communicative acts, communication theory primarily belongs to the humanities and is not a natural science. It is not a question of explanation, but of interpretation. “*As a result, ’communication theory’ will be understood as an interpretative discipline (in contrast to, for example, ’information theory’ or ’computer science’), and human communication will be seen as an important phenomenon to be interpreted*” [294]. The decisive moment in this process is the explicit choice of a certain point of view in the sense of Nietzsche’s perspectivism and Husserl’s phenomenological determination of the intentional relationship between subject and object. Each perspective requires a different interpretative framework, clarifying certain aspects instead of others. Seen from here, communication is not secure data transmission, but an intentional act of freedom to position oneself. Human communication, and this is another fundamental aspect, is the attempt to negate death by storing information, i.e., the collective intentional production of different codes. Art and culture, but above all science, including biology, genetics and the knowledge about inheritance and transformation, must be regarded as a potent method of coping with contingency, or as stated by Flusser [294]: “*Human communication is an artifice whose intention is to make us forget the brutal senselessness of a life condemned to death*”.

With regard to the phenomenon of biological heredity this applies in particular to the predictability, controllability and manipulability of the inheritance of (small) features and (minor) differences between mother and daughter cells, parents and children, in order, if not to overcome the mortality of our body and mind, then at least to pass on our soul in the form and matter of DNA as macromolecular heredity to the next generation.

As a consequence of his communication theory, Flusser states that the alphabet would be replaced as the dominant code by techno-images [297]. This will also change the concept of space and time, because geometric space and linear time are only a matter of course for people who have grown up with texts and have been shaped by texts.

Nevertheless, it seems questionable as to whether techno-images are more powerful than texts/alphanumerical codes and post-history will rely on zero-dimensional codes, exclusively, as stated by Flusser [298]. Rather inversely, do not linear codes act invincibly and insurmountably until now and presumably will survive and resist durably in the future, also for the description of the phenomenon of biological inheritance including the construction of synthetic microorganisms? Also Hans-Jörg Rheinberger emphasized the role of texts/alphanumerical codes: “*All being as being there is written being*” [299]? This apparent dualism between thinking and acting in one- vs. zero-dimensional codes might only be overcome with the development of novel x-dimensional codes exhibiting both transparency and clarity. Those could not be derived yet from the four- to zero-dimensional codes along the “4-3-2-1-0 countdown” by abstraction and reduction. Rather, they will rely on multi-perspectivity with a multitude of nuances of “colors and tones”, which, with regard to biological inheritance, transformation, and the construction of artificial microorganisms, must encompass cellular, cybernetic, topological, macromolecular heredity in addition to that of DNA. Those x-dimensional codes might enable communication or heredity as “semi-fluid” (in space) and “flowing” (in time) dimensions that lack concrete contours or sharp boundaries, at least. However, they may nevertheless defy postmodern arbitrariness and interchangeability by maintaining a liquid-crystalline state, in analogy to the separation in space-time of cells and (sub)cellular structures from neighboring counterparts as well as from one another by biological membranes during the phenomena of cellular heredity and biogenesis of organelles.

(vii) Transdisciplinary “Membranology” for Science, Literature, and Politics. In her book “Membranes: Metaphors of Invasion in Nineteenth-Century Literature, Science, and Politics”, the US biochemist, neuroscientist, comparative literature scholar, novelist, and singer-song writer Laura Otis is concerned–almost obsessed–with difference, similitude, and the boundary between those areas [300]. While witnessing a biochemical experiment studying the interaction between the eye and the neurons of the brain, Otis appreciates the necessity of change and differentiation in order for vision to work. Whether the scientific study of vision or the philosophical investigation of language, Otis concludes, difference is the common denominator [300]: “*Like our visual system, we create meaning only through the difference we perceive and the boundaries we believe are present*”. Ironically, while her experiences in both classrooms underscore the vital role of difference in the production of knowledge, they simultaneously highlight a similitude that invites the disciplinary border-crossings that Otis’ investigation enacts. The necessity for difference, in other words, provides a common ground upon which Otis constructs a provocative argument for the fundamental similitude that it masks.

In her book, Otis examines the broad social and political implications of scientific advancements in both germ and cell theories. Echoing Michel Foucault’s “The Birth of the Clinic” [301], Otis identifies the “Individual” as the focal point at which these various discourses would converge during the 19th century. With a growing focus on the cell as the locus of disease and on microorganisms as its cause, as well as on inheritance as the biological basis for susceptibility of disease transmitted along generations, scientists re-located their gaze from external environments to the internal space in which cells exist and interact. While “miasma” theory located disease with vapors that circulated within particular environments or amongst certain people, germ theory allowed disease, as manifested in microorganisms and their DNA, to penetrate the body and, thus, threaten its borders and cellular membranes via one or several of the following molecular mechanisms, such as infection, transfection, transformation, transduction.

Otis emphasizes the scientific models that motivated her analysis, suggesting that while cell and germ theories of inheritance reinforced nineteenth-century concerns with social, political, economic, and national borders, in the late twentieth century, biology offers the neuron, with its “dynamics and plasticity, [its] ability to form new connections and associations” [300], and one is tempting to add each cell, with its potency of cellular, cybernetic, topological, macromolecular and DNA heredity to create (major) features and similarities, but also (minor) differences and deviations in succeeding bacterial or unicellular eukaryotic natural or artificial cells. Can the neuron and microorganisms, she and we may ask, respectively, provide the new metaphors for a twenty-first-century society focused not on difference, but on “celebrating global connectedness” [300]?

### 4.2. “Facilitated Variation”

The creation of novel microorganisms displaying altered morphology and physiology under maintenance of their functional integrity, which represents the basis of the hypothetical Griffith transformation experiment (design 2.0), resembles the conception of “weak linkage” and “facilitated variation”, as had initially been introduced by John Gerhart and Marc Kirschner [302,303]. This concept provides an explanation for the evolution of animals under conservation of their physiological integrity and vitality (for a review, see Ref. [304]). As stressed by Gerhart and Kirschner [303], the important point is that core components and mechanisms are coupled by weak linkages, which support their rearrangement to produce new anatomical and physiological traits throughout evolution. Weak linkage “*pervades development and physiology*”, thereby abolishing the requirement for “*multiple complex instructions and precise stereochemical complementarity*” to be fulfilled by the inducer. Consequently, this enables the emergence of novel features, making weak linkage [302] “*the most important biochemical and cellular strategy used in biology, and the most unique to it*”. Accordingly, core components and molecular mechanisms engaged in the development of embryos, such as the biogenesis of organelles, membranes, cytoskeleton, regulated secretion, signal transduction, and metabolism, are connected by and operate through weak linkages. Those structures and mechanisms are therefore easily prone to reconfiguration and rearrangement. This provides the basis for the production of phenotypic variation and the emergence of novel features.

Ultimately, “facilitated variation” relies on the groundbreaking ideas of Edward E. Just’s conception of “independent irritability” [305] (“*The egg as a living cell is self-acting, self-regulating and self-realizing–an independently irritable system…like many other living cell–nerve or muscle, for example–possesses…the full capacity for development*”) and Johannes Holtfreter’s conception of “autoinduction” [306] (“*The results indicated that the treatments merely operated like an unspecific trigger, setting in motion a preexisting, pent-up mechanism, which, through unknown chain of events, led to neural differentiation*”). The marine biologist and cytologist, and embryologist, respectively, argued in their pioneering studies that the “competence” of the responding cells, organs or tissues is critical for the induction of early development, with the instructive power provoked by the inducing signal being rather marginal. Competent cells are apparently prepared for an adequate action rather than a reaction. They seem to follow selected and specific cell-intrinsic pathways under the minimal guidance of the inducing signal. Thus, competent cells are capable of specifically responding to a multitude of non-specific (and rather simple) signals in the course of selecting a certain predetermined fixed (signaling) pathway [307,308].

In an effort to reconcile the conceptions of “facilitated variation”, “rhizene agency”, “communicology” and “transdisciplinary membranology” for a more adequate understanding of inheritance in both eukaryotes and prokaryotes, “membrane landscapes” (see above) are postulated to operate as the core components together with the molecular mechanisms for their biogenesis that are connected by weak linkage through a rhizene, alphanumerical codes and porous membranes, respectively. The structural and cybernetic information required for their biogenesis and function is transferred from mother or egg cells to daughter cells along cell division (vertical inheritance) or via the release and fusion of EV or PM/OM vesicles or micelle-like GPI-AP or LP/LPS complexes (horizontal and vertical inheritance). All these mechanisms thereby cause “poising” of the daughter cells to follow certain daughter cell-intrinsic pathways upon minimal instruction by a simple, non-specific trigger. This trigger would be represented by environmental factors or, alternatively, by materials concomitantly transferred from donor to acceptor cells, such as GPI-AP, LP, integral and peripheral membrane proteins, cytoskeletal and cortical proteins arranged in micelle-like complexes or vesicles. Subsequent incorporation of those materials into the weakly linked “membrane landscapes”, which thereby display exquisite sensitivity towards “facilitated variation”, may provoke their rearrangement and reconfiguration. These molecular processes may be initiated several times in a consecutive fashion with accompanying limited adjustments, thereby leading to mixing and fitting of the core components (of “membrane landscapes”), and result in distinct and differential outputs. A key prerequisite for this intermingling and matching is the lack of an instructive nature of the signal, i.e., signal and response are not coupled, and the signal initiates latent independent pathways that pre-exist in the daughter or acceptor cells.

Research on the phenomenon of transdifferentiation, which refers to the conversion of one differentiated cell type, e.g., fibroblasts, to a different differentiated cell type, e.g., adipocytes, has raised doubts about whether [309] “*the differentiation potential of different cell types can still be allocated to a hierarchic model…rather than a flat system in which pluripotency merely represents one of many equally attainable states*”. In fact, transdifferentiation argues for the validity of a “primus inter pares (first among equals)” model, which may be perfectly represented by the “rhizene agency”, the countdown of the codes of communication and the exchange between porous membranes. It does not lead to preference for one of the alternate cell fates, i.e., fibroblast vs. adipocytes, but rather presents them “*as dents along the circumference of a flat disk*” [308]. In eukaryotes as well as prokaryotes, the conversion of one (morphological and/or physiological) fate to another one may be provoked by the torsion of a disk-like “membrane landscape” at a certain angle in a specific direction in response to certain environmental factors, such as mechanic distortion. Alternatively, the incorporation of materials, such as (lyso)phospholipids, cholesterol, LPS, GPI-AP, LP, transmembrane and peripheral, cytoskeletal and cortical proteins, which are typically assembled in micelle-like complexes, might be transferred into “membrane landscapes” as one of the core component that manage to induce alteration of cell fate. Thereby, the converted, i.e., tilted state of the disk or “membrane landscape” may be stably maintained. Alternatively, it may be attenuated and then gradually become lost from that state, with accompanying reversion of the original fate. Thus, distinct activation “states” seem to be coupled by weak linkage, and the transformation of one state to another could be elicited by environmental factors as a non-instructive inducer or signal.

A similar explanation may hold true for the phenomenon of phenotypic plasticity, which is based on the selection of varying hidden developmental pathways depending on existing environmental conditions faced by a cell. This selection necessitates the capability of producing extended phenotypic changes that “*already exist in the organism in self-inhibited alternative states, and (one or another of) these can be elicited by simple signals*” [304]. Such a “selection” procedure, rather than mere “pressure” by certain environmental factors, may ultimately lead to pronounced changes during evolution, even in the absence of any alterations in the (epi)genome (for a review, see Refs. [291,309,310,311]).

Finally, the conceptions of “materialistic organicism”, “facilitated variation”, “rhizome/rhizene agency”, “communicology” and “transdisciplinary membranology” as have been introduced by Just, Gerhart/Kirschner, Deleuze/Guattari, Flusser, and Otis, respectively, are aimed at reconciling vitalistic and reductionistic positions for an explanation of the emergence of the increasing complexity and variation in the existing life world. Again, they reflect current and ongoing efforts to overcome the dichotomies and agential cuts which are still prevalent in the scientific explanation and sociocultural interpretation of “natural” and “constructed” phenomena, such as biological inheritance and the creation of synthetic microorganisms, by the natural sciences and the philosophy of science or sociology of knowledge, respectively. Only the above conceptions, sometimes collective called “molecular vitalism” [302] and positioned between those of “holism” (Jan Smuts [312]), “Gestalt theory” (Cesar Koppe Grisolia [313]), and “creative and morphogenetic vitalism” (Alfred North Whitehead [314] and Rupert Sheldrake [315], respectively) on the one side and “ontological and epistemological reductionism” (Richard Jones [316] and Hilary Putnam [317], respectively), mechanistic causality (Raffaella Campaner [318]) and “unified science” (Otto Neurath [319]), on the other side will manage to overcome the limitations of our past and current efforts aimed at the creation of novel microorganisms. Those have been set by the history of organic life on earth and the continuity of cellular heredity in its wholeness or “Gestalt” from the “first or single ancestral cell” to the age of synthetic biology. It is tempting to speculate that by using the strategies of the above conceptions, the construction of novel microorganisms will be easier to tackle compared to the modification of other typical human agencies which have been formed as a consequence of socio-cultural traditions in the course of mankind.

The final statement on the possibility of creating novel (micro)organisms dealt with in this review deserves Jacques Loeb with an apparently unexpected statement in view of his above-mentioned opinion [320]: “*Without a structure in the egg to begin with, no formation of a complicated organism is imaginable…*”.

## Figures and Tables

**Table 1:** Design of and consequences from the Griffith transformation experiments 1.0-2.X. The various designs, which either had already been performed (1.0–1.3) or still remain to be conducted (1.4-2.X, tbd), are compiled with regard to the experimental system and fractionation procedure used, the phenotype analyzed, the detection method employed, and the representative authors involved. The nature of the entities and principles transferred from donor to acceptor bacteria and the underlying conceptions are given. The inherited phenotypes, which are explained best by the various experimental designs, are indicated as are the intentional foundations. Boxes of different colors indicate the entities transferred along, following the various designs. +, concerted action between shape/information and substance/matter.

Experiment	Model	Fractionation	Analysis	Detection	Author	Transferred Entities	Transferred Principle	Theory of Inheritance	Inherited Phenotype	Intention
Griffith 2.1-2.X	Homo- genate	- Centrifugation (high speed) - Filtration - Purification	- Morphology - Complete Metabolic Pathways - Overall Architecture	- Imaging - Proteomics - DNA Sequencing	tbd	- OM-Vesicles - Micelle-like LPS Compl - Cytoskeleton - Membranes	- Shape/Information + - Substance/Matter	- Extended - Plasmon - “Soft“ - Rhizene	- Major Features - Large Differences - Statics - Pathways - Building Plans - Cybernetics - Dynamics	- Novel Cells
Griffith 2.0	Cells	- none				- Metabolites - Feedback loops - Regulatory Circuits - Turnover				
Griffith 1.4-1.X	Homo- genate	- Phospholipase - Centrifugation (high speed)	- Resistance - Auxotrophy	- Selection	tbd	- Prions - Int Disord Prot - OM-Vesicles - Micelle-like LPS Compl	- Substance/Matter	- Including - Holistic	- Minor Features - Small Differences	- Mode of Inheritance
Griffith 1.3		- Chloroform - Centrifugation - Nuclease - Protease - Water-Sol. Fr.	- Virulence	- Microscopy - Serotypes	Avery MacLeod McCarty Alloway	- DNA	- Shape/Information + - Substance/Matter	- Centric - Excluding - “Hard“		- Novel Cells - Matter of Inheritance
Griffith 1.2		- Heat - Soluble Fract.		- Serotypes - Lethality Mice	Dawson					
Griffith 1.1	Mouse	- Heat			Griffith Neufeld Levinthal					
Griffith 1.0		- none			Griffith					- Bacterial Variability - Infections

**Table 2:** Ranking of the various experimental systems considering their configuration (exclusion index E) and method of detection (bias index B). Experimental systems for “in vivo blending” are classified according to co-injection of acceptor bacteria (*S. pneumoniae*) together with the putative transforming principle derived from (*S. pneumoniae*) total cells, cell-free homogenate, non-DNA matter, or DNA into mice. Alternatively, experimental systems for “in vitro transformation” are classified according to direct incubation of acceptor bacteria with the putative transforming principle as above. The experimental systems which had previously been used (X) or remain to be done in the future (tbd) are evaluated for both the specific configuration and elaborated detection method, with the exclusion indices E for in vivo “blending” set at 1 for cells, 2 for homogenate, 3 for non-DNA matter, 4 for DNA, and in vitro transformation set at 3 for cells, 4 for homogenate, 5 for non-DNA matter, 6 for DNA and the bias indices B set at 1 for morphology/physiology, 2 for virulence (only in vivo “blending”), 3 for serotype/capsule. The total sum T is calculated as the sum of the corresponding exclusion and bias indices E and B, respectively. The relative ranking R is given with the lowest (green) and highest (red) T, respectively, for each experimental system and detection method.

**Configuration**		Ranking of Various Experimental Systems							
		In Vivo “Blending”				In Vitro Transformation			
	Host	Mice				-			
	Acceptor	*Streptococcus pneumoniae*							
	Transforming Principle	Cells	Homo- genate	Non-DNA Matter	DNA	Cells	Homo- genate	Non-DNA Matter	DNA
	Design	2.0	2.1	2.2-2.X	1.3	1.0	1.2	1.4-1.X	1.3
**Detection Method**	Virulence in Mice	X	X	tbd	X	not relevant			
		E1/B2	E2/B2	E3/B2	E4/B2				
		T3/R2	T4/R3	T5/R4	T6/R5				
	Capsule Serotype	X	X	tbd	X	X	X	tbd	X
		E1/B3	E2/B3	E3/B3	E4/B3	E3/B3	E4/B3	E5/B3	E6/B3
		T4/R3	T5/R4	T6/R5	T7/R6	T6/R5	T7/R6	T8/R7	T9/R8
	Morphology Physiology	tbd	tbd	tbd	not relevant	tbd	tbd	tbd	tbd
		E1/B1	E2/B1	E3/B1		E3/B1	E4/B1	E5/B1	E6/B1
		T2/R1	T3/R2	T4/R3		T5/R4	T5/R4	T6/R5	T7/R6

## Abbreviations

The following abbreviations are used in this manuscript:

LP	Lipoprotein(s)
LPS	Lipopolysaccharide(s)
OM	Outer membrane(s)
PEG	Polyethyleneglycol
PM	Plasma membrane(s)
(R)/(S)	Rough/Smooth pneumococcal serotype
STS	Science and technology studies
